# Endangered but genetically stable—*Erythrophleum fordii* within *Feng Shui* woodlands in suburbanized villages

**DOI:** 10.1002/ece3.5513

**Published:** 2019-09-10

**Authors:** Zheng‐Feng Wang, Hai‐Lin Liu, Se‐Ping Dai, Hong‐Lin Cao, Rui‐Jiang Wang, Zhang‐Ming Wang

**Affiliations:** ^1^ Center of Plant Ecology, Core Botanical Gardens Chinese Academy of Sciences Guangzhou China; ^2^ Guangdong Provincial Key Laboratory of Applied Botany, South China Botanical Garden Chinese Academy of Sciences Guangzhou China; ^3^ Environmental Horticulture Research Institute Guangdong Academy of Agricultural Sciences Guangzhou China; ^4^ Key Lab of Ornamental Plant Germplasm Innovation and Utilization Guangzhou China; ^5^ University of Chinese Academy of Sciences Beijing China; ^6^ Guangzhou Institute of Forestry and Landscape Architecture Guangzhou China

**Keywords:** bottleneck, demographic history, genetic diversity, microsatellites, parentage analysis

## Abstract

*Feng Shui* woodlands are naturally or artificially formed green areas in southern China. They are precious for maintaining ecosystem balance in modern semiurban environments. However, they are generally small and geographically isolated from each other, and the status of genetic diversity of the plant species within them has been almost neglected. Therefore, we studied the genetic diversity of the endangered *Erythrophleum fordii* in eight *Feng Shui* woodlands (a total of 1,061 individuals) in Guangzhou, a large city in southern China, using microsatellites. For comparison, one population with 33 individuals sampled in a nature reserve was also studied. Although our results indicate that significant demographic declines occurred historically in *E*. *fordii*, such declines have not resulted in consistent reductions in genetic variation over generations in *Feng Shui* populations in the recent past, and the levels of genetic variation in these populations were higher than or comparable to the genetic variation of the population in the nature reserve. In addition, our parentage and paternity analyses indicated widespread and potential long‐distance pollen flow within one *Feng Shui* woodland, indicating the presence of an unbroken pollination network, which would at least partially alleviate the genetic erosion due to habitat fragmentation and the unequal gene contributions of *E*. *fordii* parents to their progenies when favorable recruitment habitats are absent under most of the parent trees. Overall, our results suggest that *E*. *fordii* in *Feng Shui* woodlands may not be driven to extinction in the near future. Nevertheless, uncontrolled fast urban development with a lack of awareness of *Feng Shui* woodlands will cause the local extinction of *E*. *fordii*, which has already happened in some *Feng Shui* woodlands.

## Graphical abstract

It is the first report about genetic sustainability of tree species under urbanization and habitat fragmentation in China. The results will be combined with the one of the other regions to give a general conclusion of tree species' genetic health under urbanization and guide not only *Feng Shui* woodlands conservation but also urban reforestation to balance species conservation and economic developments.

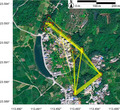

## 1 INTRODUCTION

Culturally protected forests (CPFs, Hu, Li, Liao, & Fan, 2011) are formed through religious or traditional belief (Avtzis et al., 2018). They are important rural/urban forest types worldwide including Asia, Africa, Europe, and Latin America. They are known as *Feng Shui* woodlands in China (Ye, Xu, Wu, & Cao, 2013) and sacred groves (Avtzis et al., 2018; Bossart & Antwi, 2016) and village groves (Lee, Hong, & Kim, 2019) in the other countries. They are generally small in size but can serve as refuges for a large number of regional species (Avtzis et al., 2018; Bossart & Antwi, 2016; Hu et al., 2011; Lee et al., 2019; Martinez & Amar, 2014).

*Feng Shui* woodlands have existed in China for more than 2000 years (Coggins, 2003; Guan, 2002; Hu et al., 2011; Ye et al., 2013). Literally, “*Feng*” means wind and “*Shui*” means water in Chinese. Following these definitions, *Feng Shui* is thought to create harmony, and promote health and wealth in indigenous communities. In addition to maintaining biological diversity, they are valuable for regulating the climate, cleaning the air, and protecting soil and water, and they play important roles in cultural heritage, leisure activities, and the local economy.

*Feng Shui* woodlands can be categorized into three types according to their location: village, cemetery, and temple *Feng Shui* woodlands, and among them, village *Feng Shui* woodlands are the most common (Hu et al., 2011; Ye et al., 2013). With the recent development and expansion of cities in southern China, some of these woodlands have become part of cities, such as Guangzhou, which is an ever‐growing megacity in southern China. Currently, there are 156 *Feng Shui* woodlands in and around Guangzhou city with a total of area 521.07 ha (Ye et al., 2013). However, because most of these are not included in the local conservation projects of the city government, relentless construction (such as housing developments and road construction) in the city is a constant threat to the woodlands, shrinking their areas and isolating them more from each other. Thus, urbanization contributes greatly to habitat destruction and fragmentation, altering the plant and animal communities of *Feng Shui* woodlands.

The negative effects of urbanization on species genetic diversity have been documented in depth (Johnson & Munshi‐South, 2017; Johnson, Thompson, & Saini, 2015) and investigated worldwide (Bartlewicz, Vandepitte, Jacquemyn, & Honnay, 2015; Dubois & Cheptou, 2017; Hermansen, Roberts, Toben, Minchinton, & Ayre, 2015; Nagamitsu, Kikuchi, Hotta, Kenta, & Hiura, 2014; Vranckx et al., 2014; Wang, Sork, Wu, & Ge, 2010). However, in a review of such effects on tree populations, it was found that they do not automatically lead to the extinction of tree species on a large scale. Prolonged clonal growth, long generation times, and long‐distance pollen dispersal help species temporarily escape or postpone extinction (Honnay & Bossuyt, 2005; Low, Cavers, Boshier, Breed, & Hollingsworth, 2015), which results in an undetectable erosion of plant species genetic variation, making it less likely for people to start immediate conservation efforts.

So far, only two plant genetic diversity studies in *Feng Shui* woodlands in China have been carried out (Ge, Liu, Shen, & Lin, 2015; Wang, Ye, Fu, Ren, & Peng, 2008), and their results generally confirmed the results of Honnay and Bossuyt (2005) and Low et al. (2015) and clearly demonstrate the harmful effects of urbanization on species' genetic health. In the work of Wang et al. (2008), they compared the genetic diversity of a common species *Cryptocaya chinensis* in two *Feng Shui* woodlands and four natural reserves in the lower subtropical region of southern China. Their results revealed unexpected extensive clonal growth of *C. chinensis* in two *Feng Shui* woodlands due to severe fragmentation and the small population sizes in suburban areas. The clonal growth in *Feng Shui* woodlands maintained a substantial proportion of the genetic variation of the initial populations, and the small sizes of the woodlands did not result in significant genetic differentiation from the larger reserve populations. However, as McDonald, Rice, and Desai (2016) found out that asexual population maintained genetic variation at the cost of fixing substantially deleterious mutations while sexual population allowed natural selection to more efficiently sort beneficial from deleterious mutations and speeded adaptation, extensive clonal growth may threaten the long‐term adaptation of *C. chinensis* in *Feng Shui* woodlands. In the work of Ge et al. (2015), they compared the genetic diversity of *Phoebe bournei* in three *Feng Shui* woodlands and three natural reserves in southern China. Their results showed that the genetic diversity of *P*. *bournei* was clearly lower in *Feng Shui* woodlands than in the reserves, which could be related to its low regeneration rate in *Feng Shui* woodlands.

Given that some of plant species in *Feng Shui* woodlands are even endangered, studies on their conservation are thus needed to improve our ability to make relevant recommendations on ways to alleviate the negative impacts of urban development on native species. Therefore, our objectives were to study the genetic diversity and within population gene flow of the endangered species *Erythrophleum fordii* in *Feng Shui* woodlands in Guangzhou, China.

Due to severe human disturbance and the small population size, we would expect the genetic diversity of *E. fordii* in *Feng Shui* populations to have decreased. Furthermore, because gene flow is crucial to maintain genetic diversity in plant populations, by examining the present gene flow pattern via parentage analysis, we aimed to investigate the relationship between genetic diversity and gene flow patterns. Currently, there are contrasting results regarding such a relationship in the literature. Theoretically, human disturbances change pollen mutualisms in *Feng Shui* woodlands and, subsequently, alter a population's genetic variation. However, empirical evidence does not always support this in other disturbed habitats (Giombini, Bravo, Sica, & Tosto, 2017; Noreen, Niissalo, Lum, & Webb, 2016; Rosas, Quesada, Lobo, & Sork, 2011). Therefore, the genetic consequences for a small population or due habitat disturbance are both species and location specific (Owusu, Schlarbaum, Carlson, & Cailing, 2016; Schwarcz et al., 2018). In particular, we also examined the demographic history of *Feng Shui* populations of *E. fordii* to study how the current level genetic diversity is related to historical events such as bottlenecks. As a valuable timber tree, the endangered status of *E. fordii* is believed mainly to be the result of large‐scale logging in the past. Consequently, it will leave a clear signal of a population decline in genetic diversity.

## 2 MATERIALS AND METHODS

### 2.1 The species

Zhu, Wang, Ye, and Cao (2013) and Zhu, Wang, Ye, Cao, and Saravanan (2013) have described *E. fordii* in detail. Briefly, *E. fordii* is a legume species, belonging to the family Fabaceae. It occurs naturally in China and Vietnam. Due to the hardness of its wood, it is commonly known as the “ironwood” tree in China. In the past, overexploitation has made it endangered in the wild (IUCN^1^). At present, it is under second‐class national protection in China. It is a typical outcrossing species with pollen dispersed by many kinds of insects, such as beetles, butterflies, bees, and wasps (Zhu et al., 2013). However, self‐fertilization could happen in *E. fordii* (Zhu et al., 2013), and it was also reported in the congeneric species *E. suaveolens* (Duminil et al., 2016). Its seed is flat and ovate‐shaped, large and heavy with 1.54–1.67 cm long, 1.28–1.43 cm wide, 0.784–0.917 g in weight (Zhao et al., 2009). It is inedible and has no wing and no particular attributes to attract animals; therefore, it is believed to be dispersed by gravity (Zhu et al., 2013).

### 2.2 Sample collection

**Figure 1:**
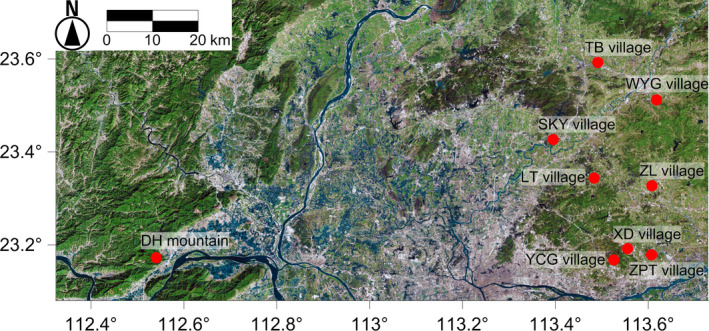
Map showing the locations of the sampled populations (the background layer was downloaded from Google Maps in this and all the other figures). The full names of the populations are given in Table 1

According to Ye et al. (2013), *E. fordii* can be found in more than 30 *Feng Shui* woodlands in Guangzhou city. By comparing their geographic locations and population sizes, we sampled eight of them (Table 1, Figure 1, Figure S1) during 2017 and 2018. During sampling, we carefully examined within and around each population to make sure we would not miss *E. fordii* populations or individuals nearby. For comparison, one population from Dinghu Mountain Nature Reserve (DH Mountain) in Zhaoqing city, China, was also sampled. For convenience, we will use the abbreviations of the village names (Table 1) for the populations. The pairwise geographic distances between populations are shown in Table S1.

**Table 1:** Studied populations of *Erythrophleum fordii* and their genetic diversities

Populations in different places and their abbreviation^a^	*N*	*A*_R_	*A*_P_	*H*_O_	*H*_E_	*f*	Sampling year
Tangbei (TB) village	346	3.2224	0.1717	0.4999	0.4832	−0.0346*	2017
Adult	18	3.1377	0.2318	0.5058	0.5000	−0.0120	
Juvenile	71	3.0376	0.2121	0.5157	0.4903	−0.0522*	
Seedling	257	2.8843	0.1033	0.4951	0.4756	−0.0412*	
Wayaogang (WYG) village	60	2.8472	0.0268	0.4632	0.4007	−0.1647*	2018
Adult (6) + Juvenile (12)	18	2.4845	0.2345	0.4561	0.4007	−0.1431*	
Seedling	42	2.6140	0.3639	0.4704	0.4008	−0.1757*	
Liantang (LT) village	130	3.2493	0.0475	0.4940	0.4865	−0.0134	2018
Adult (9) + Juvenile (75)	84	3.3737	0.3391	0.5063	0.4929	−0.0272*	
Seedling	46	3.3084	0.2738	0.4817	0.4801	−0.0033	
Zhongtou (ZPT) village	164	3.2691	0.0640	0.4995	0.4774	−0.0509*	2018
Adult	59	3.1118	0.0852	0.4888	0.4520	−0.0822*	
Juvenile	27	3.2538	0.1350	0.4893	0.4852	−0.0086	
Seedling	78	3.2099	0.1132	0.5202	0.4950	−0.0513*	
Zhongling (ZL) village	82	3.1667	0.0605	0.5452	0.4982	−0.0958*	2018
Adult	65	2.8857	0.2218	0.5425	0.4904	−0.1072*	
Juvenile (5) + Seedling (12)	17	2.9813	0.3174	0.5480	0.5061	−0.0857*	
Shuikouying (SKY) village	276	3.3219	0.0592	0.5467	0.5330	−0.0285*	2018
Adult	155	3.3291	0.1015	0.5474	0.5348	−0.0236	
Juvenile	34	3.2169	0.0435	0.5310	0.5262	−0.0091	
Seedling	87	3.3611	0.1158	0.5617	0.5379	−0.0446*	
Yangchengang (YCG) village	1	−	−	−	−	−	2018
Xiaodong (XD) village	2	−	−	−	−	−	2018
Dinghu (DH) Mountain	33	3.1533	0.3089	0.4631	0.4518	−0.0244	2018
Adult	33	3.1533	0.3089	0.4631	0.4518	−0.0244	
Overall	1,094			0.5125	0.5461	0.0615*	

Abbreviations

*A*_P_, Private allelic richness; *A*_R_, Allelic richness; *f*, inbreeding coefficient; *H*_E_, unbiased expected heterozygosity; *H*_O_, observed heterozygosity; *N*, sample size.

a The letters in parentheses show the abbreviated population name, the numbers in parentheses indicate the sample size of the life stage.

* *p* < .05 after the Bonferroni correction.

Except for SKY village, the initial colonization history of *E. fordii* (natural vs. artificial) in the other seven woodlands and DH Mountain are unknown. According to records, *E. fordii* was planted in SKY village in 1368 A.D. by a troop stationed there that used this tree to make arrows. However, in TB village, one *E. fordii* tree is tagged with a plate which estimates it to be more than 200 years old (Figure S3).

**Figure 2:**
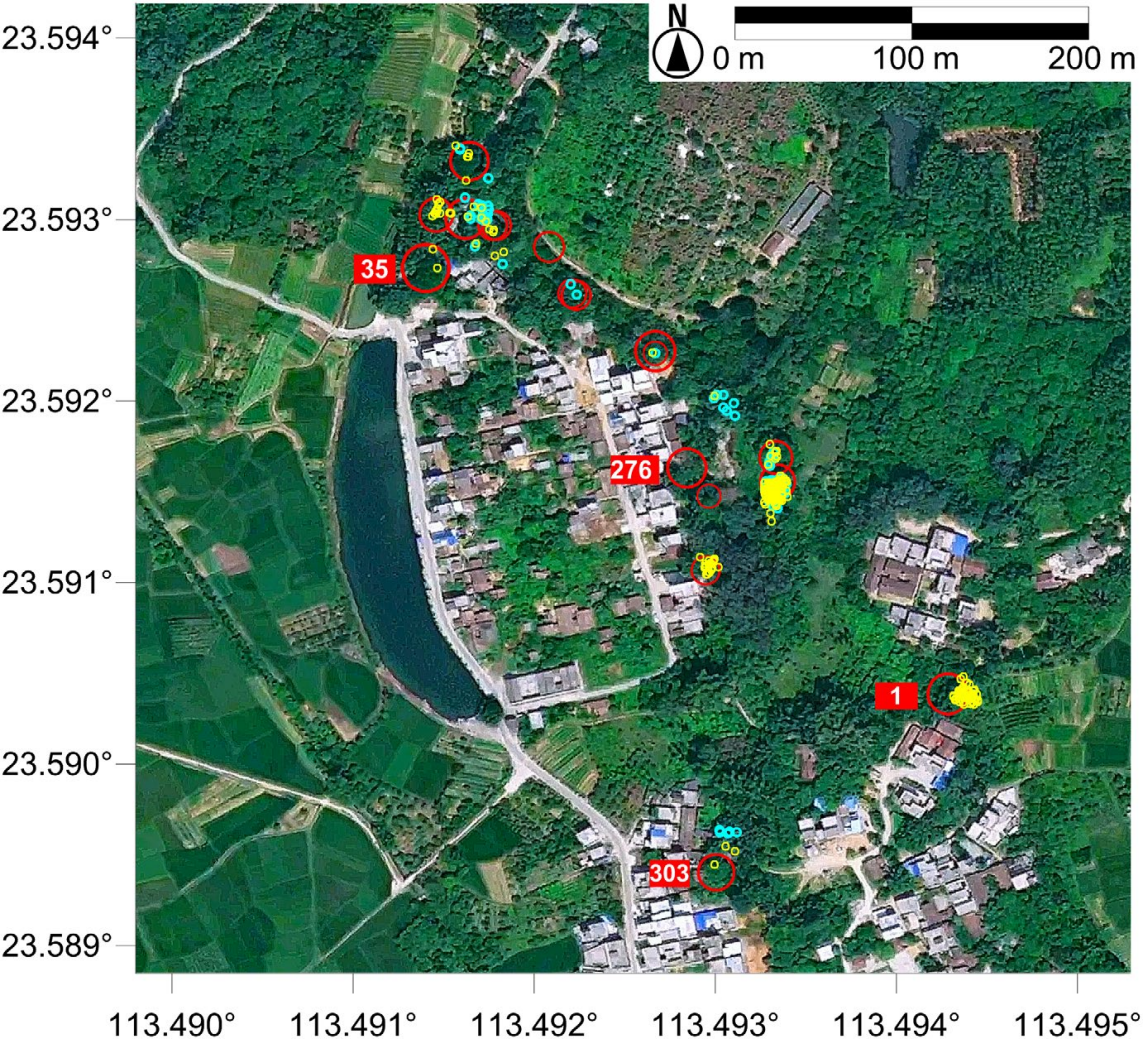
Spatial distribution of *Erythrophleum fordii* individuals in TB village in Guangzhou city, China. Red: adults; cyan: juveniles; and yellow: seedlings. Only adults are given proportional circles corresponding to their DBH values. An additional 44 seedling samples were collected from around the adult numbered 303. Since these seedlings grew on the roof of an abandoned hut beside this adult tree, their positions were not recorded by GPS (indicated in Figure S2) and therefore, these 44 seedlings are not shown on the map. According to the plate tagged by the local Forest Management Department in 2013, the individual numbered 1 is more than 200 years old (Figure S3). The photographs of the two individuals numbered 35 and 276 are shown in Figure S4

We sampled all the individuals that we could find in the populations of TB, WYG, XD, YCG, ZL, and LT villages (Figure 2, Figure S1). Unfortunately, due to human interference, the populations of YCG and XD villages, which were previously recorded as having large population sizes, had shrunk to one and two individuals, respectively. Since both ZPT and SKY villages contained hundreds of newly sprouted seedlings, which was more than we could genotype with our budget, we randomly sampled only some of the seedlings but all of the individuals with DBH (diameters at breast height) ≥1 cm or height ≥1 m.

We collected one to three leaf samples per *E. fordii* individual and put them into sealed plastic bags containing silica gels. We determined the locations of the sampled individuals using GPS, and measured and recorded their DBH if the DBH ≥1 cm, or height if the DBH <1 cm.

In April 2018, we revisited the sampling site in TB village and recorded the flowering status of the individuals. We observed that only individuals with DBH >20 cm could flower. By combining flowering status and DBH and/or height information, we classified all of our sampled individuals into three cohorts: adult (DBH ≥20 cm), juvenile (1 cm ≤ DBH < 20 cm), and seedling (DBH <1 cm).

DH Mountain was the first nature reserve in China (Zhu et al., 2013), and the *E. fordii* population in it is believed to be well preserved. According to Zhu et al. (2013), there were a total of 528 *E. fordii* individuals including 78 with DBH ≥15 cm. Therefore, we used it as the basis to compare the highly disturbed populations in the *Feng Shui* woodlands. However, similar to the populations in *Feng Shui* woodlands, it is in a small area of approximately 2 ha, although there are no obvious environmental limitations that might have prevented it from growing in the surrounding areas (personal observation, WZF). In this study, we resampled 33 individuals with DBH >30 cm and genotyped them using not only the previously isolated loci, but also our newly developed ones (Table S2).

### 2.3 Microsatellite isolation and genotyping

Since only nine microsatellites in *E. fordii* were previously isolated and characterized (Zhu et al., 2009), to increase the discrimination power in parentage analysis, we used the restriction site‐associated DNA sequencing (RAD‐seq) method to obtain some new microsatellite markers in *E. fordii*.

By using two *E. fordii* individuals from the South China Botanical Garden, we constructed two RAD‐seq libraries according to the methods described by Baird et al. (2008). Briefly, whole genome DNA of *E. fordii* was digested using the restriction enzyme *Eco*RI (Takara). The digested DNA fragments were then ligated to adaptors and PCR amplified. Approximately 300–500 bp fragments were subsequently selected and sequenced on Illumina HiSeq X Ten genetic analyzer (Illumina) to produce 150 bp paired end sequencing reads. After sequencing, we obtained a total of 49,971,692 bp of raw reads for one individual and 39,494,352 bp for the other. The raw sequence data are available in the NCBI SRA database with accession numbers SRX5010692 and SRX5010693. Filtering for PCR duplicates and low‐quality reads resulted in 16,662,656 and 10,993,602 bp of useful reads for the two individuals, respectively. These reads were assembled using Rainbow 2.0.4 (Chong, Ruan, & Wu, 2012), and the assembled contigs were combined and re‐assembled using CAP3 (Huang & Madan, 1999). We used Msatcommander 0.8.2 (Faircloth, 2008) to screen for microsatellites in the re‐assembled contigs. In particular, we only chose sequences with at least eight and seven dinucleotide and trinucleotide motifs repeats for the two individuals, respectively. We then randomly chose 35 microsatellite sequences to perform PCRs to test their availability.

We followed the PCR procedures described by Zhu et al. (2009) but with an annealing temperature of 53°C for all the microsatellite loci. We ran the PCR products on 2% agarose gels which revealed that 29 microsatellites could be successfully amplified to produce fragments of the correct size. We then used six individuals from TB village to perform PCRs to study the polymorphism of the 29 microsatellites. After PCR amplification and running the PCR products on an ABI 3730 sequencer, we identified 16 polymorphic microsatellites with clear electrophoretic profiles of alleles in the six individuals. Using these six individuals, we also tested the seven microsatellites previously isolated by Zhu et al. (2009) and shown to be in Hardy–Weinberg equilibrium (HWE). However, only five of these could be successfully amplified in all of our present samples. Thus, we used these 5 together with the 16 newly identified microsatellites (Table S2) to genotype 346 individuals from TB village. We tested the HWE of the microsatellites and found that the locus *EF‐32* not only showed a significant deficit in heterozygosity at the population level, but also in the seedling life stage (Table S3). Therefore, we did not use this locus in our study to avoid null allele errors and instead used a total of 20 loci for all population genotyping and data analyses.

### 2.4 Data analysis

Since only one and two *E. fordii* individuals were found in YCG and XD villages, respectively, these three individuals were only used to estimate the overall genetic diversity in *E. fordii* but excluded from the other data analyses.

We first estimated null allele frequencies at 20 loci in each population with INEST v2.2 under the individual inbreeding model (IIM) with default parameters (Chybicki & Burczyk, 2009). IIM was implemented by a Bayesian approach which showed better statistical properties than maximum likelihood and the other approaches (Chybicki & Burczyk, 2009). We then calculated genetic diversity parameters, observed and unbiased expected heterozygosity (*H*_O_, *H*_E_) using GenAlEx 6.501 (Peakall & Smouse, 2012), and the inbreeding coefficient (*f*) using GENEPOP 4.3 (Rousset, 2008). Since the number of alleles depended on the sample size, which made the allelic richness (*A*_R_) and private allelic richness (*A*_P_) results difficult to compare among populations or life stages, we used the ADZE 1.0 program (Szpiech, Jakobsson, & Rosenberg, 2008) to compute rarefied allelic richness and private allelic richness by controlling for the smallest sample sizes. That is, for population comparison, we computed *A*_R_ and *A*_P_ using the smallest sample of DH Mountain (*N* = 33), while for life stage comparison within population we computed *A*_R_ and *A*_P_ using the smallest sample of life stages in each population. Nonparametric Wilcoxon tests were used to test for differences in diversity estimates (*H*_O_, *H*_E_, *A*_R,_ and *A*_P_) between populations and life stages within population. The Wilcoxon tests were performed with *wilcox.test* function in R software and one‐sided *p*‐values (“less” or “greater”) were reported.

We also used GENEPOP 4.3 to assess the deviation from HWE and genotypic linkage disequilibrium (LD) among all pairs of loci. The levels of significance for HWE and LD were adjusted by using the sequential Bonferroni correction (Holm, 1979). At the population level, since the association analysis between loci via the LD tests may be strongly influenced by any family structure (Flint‐Garcia, Thornsberry, & Buckler, 2003) present in our data, we only used adults or adults and juveniles (in WYG and LT villages) in the LD tests for our studied loci. At locus *Gm2024*, some individuals produced abnormal alleles whose sizes did not follow the rule for the gain or loss of repeated unit; therefore, we treated these alleles as missing values for those individuals in subsequent bottleneck and demographic history inference analyses, but not for other analyses.

Since only TB village was sampled thoroughly, especially for seedlings, we only performed a parentage analysis in this population using the Cervus 3.07 program (Kalinowski, Taper, & Marshall, 2007). Before performance parentage analysis, the power of exclusion for the microsatellite loci was estimated by Cervus. The cryptic gene flow was then assessed by 1−(1−*P*_parent‐pair_)^Na^ following Dow and Ashley (1996), where *P*_parent‐pair_ was combined nonexclusion probability of parent pair, and Na was the number of adults used for parentage analysis (18 in this study). After determining the power of exclusion for the microsatellite loci, using the allele frequency data calculated from all the samples in this population, we ran a simulation to estimate the critical Delta scores necessary for parentage assignments at a 95% confidence level. The simulation parameter values were as follows: 100,000 tests, 18 for the candidate parents (the adults in our samples of TB village), 0.9 for the proportion of candidate parents sampled, 1 for the proportion of loci genotyped, 18 for the minimum genotyped loci, self‐fertilization was allowed, and the default setting were used for the other parameters. Although we could identify the parents at a 95% confidence level for most of the seedlings, there were some seedlings that we could not identify the parents of. Considering the restricted seed dispersal ability of *E. fordii* for the rest of the seedlings, we assumed the mother tree to be the nearest tree to the seedlings geographically, and then used the same program to perform paternity analysis. In this analysis, we used the same simulation parameters as in the above parentage analysis. After parentage and paternity analyses, the actual pollen immigration rate was estimated as number of seedlings with undetermined paternity/total number of seedlings. The effective pollination neighboring area (*A*_ep_) was calculated by *A*_ep_ = 2*πσ* ^2^, where *σ* ^2^ was the variance of the pollen dispersal distance (Levin, 1988).

We also estimated the pollen immigration using the spatially explicit neighborhood model (Burczyk, Adams, Birkes, & Chybicki, 2006) in the NM+ 1.1 (Chybicki & Burczyk, 2010) which simultaneously estimated seed immigration. Using maximum likelihood, we estimated the self‐fertilization rate (*s*), the pollen immigration rate (*mp*), pollen dispersal distance (*dp*), the seed immigration rate (*ms*), and seed dispersal distance (*ds*). If initial parameter values were far from the true values, the maximum likelihood algorithm could fail to reach convergence. After trying different initial parameters, the final parameter settings used for estimation were as follows: exponential‐power dispersal kernel for both seed and pollen, genotyping error rates 0.01 for all loci, seed immigration rate 0.01, average seed dispersal distance 4.57, shape parameter of seed dispersal kernel 0.42, pollen immigration rate 0.134, average pollen dispersal distance 160, shape parameter of pollen dispersal kernel 1.6, selfing rate 0.12, and default for the other parameters.

We used both heterozygosity‐excess (Cornuet & Luikart, 1996) and M‐ratio methods (Garza & Williamson, 2001) implemented in INEST v2.2 (Chybicki & Burczyk, 2009) to test for recent population bottlenecks. For both methods, we used Wilcoxon's signed‐rank test (10,000 permutations) under a two‐phase mutation (TPM) model in INEST v2.2 to determine the significance of the bottlenecks. The parameters for TPM were 3.1 for average size of multistep mutations and 0.22 for proportion of multistep mutations.

To infer the population demographic history, we used DIYABCskylineplot 1.0.1 (Navascués, Leblois, & Burgarella, 2017) to detect and characterize past contractions or expansions using microsatellites. This program uses coalescent theory to estimate the population size changes with generations. After initial trials, we set the following parameter values for DIYABCskylineplot analysis: num_of_points = 100 (number of points to draw skyline plot), prior_THETA_min = 0.1 (THETA, denoted by *θ*, is the population size and measured by 4*Nμ*, where *N* is the effective population size and *μ* is the mutation rate per generation), prior_THETA_max = 10, prior_GSM_min = 0.1 (GSM is the generalized stepwise mutation model for microsatellites), prior_GSM_max = 0.8, the repeat size for each locus was specified, and all other options and priors were set to default values.

To perform the bottleneck analysis and infer the population demographic history, we used only the adult individuals for the populations of ZPT, ZL, SKY villages, and DH Mountain; for the populations of TB and LT villages which contained only a few adults, we used both adult and juvenile individuals, and for the population of WYG village, the number of adult and juvenile as only 18 (Table 1); therefore, we used all of the individuals (Table 2).

**Table 2:** Bottleneck analyses in *Erythrophleum fordii* populations

Population	Number of individuals	*p*‐value based on heterozygosity‐excess method	*p*‐value based on M‐ratio method
TB	89 (Adult + Juvenile)	**.0274**	**.0020**
WYG	60 (all individuals)	.2980	**.0032**
LT	84 (Adult + Juvenile)	**.0102**	**.0003**
ZPT	59 (Adult)	**.0476**	**.0024**
ZL	65 (Adult)	**.0054**	**.0037**
SKY	155 (Adult)	**.0004**	**.0044**
DH	33 (Adult)	.2445	**.0010**

Note

The numbers in bold are *p* < .05; refer to Table 1 for the population names.

We finally examined genetic structure among populations by STRCTURE 2.3.4 (Pritchard, Stephens, & Donnelly, 2000). Assuming admixture model with correlated allele frequency, twenty independent runs were performed for each possible cluster (*K*, from 1 to 8) using a 5 × 10^5^ Markov Chain Monte Carlo (MCMC) iterations after a burn‐in period of 5 × 10^5^ on total multiloci genotypes of adults without prior concerning of their origin populations. The choice of the probable *K* value was made both as recommended in STRUCTURE user's manual and by Δ*K* method (Evanno, Regnaut, & Goudet, 2005). For the inferred *K*, we used CLUMPP 1.1.2 (Jakobsson & Rosenberg, 2007) to calculate the average membership coefficient for each individual by combing the results of 20 runs. All these analyses were performed by a combination of functions from the StrataG 2.1 (Archer, Adams, & Schneiders, 2017) in R package.

## 3 RESULTS

The number of alleles per locus varied from 2 to 11 (Table S3) among the 20 microsatellite loci studied for the populations of *E*. *fordii*, and the locus *EF‐33* had the highest number of alleles. Nineteen of the loci showed very low null allele frequencies (<0.005) in all populations, and locus *EF‐28* displayed null allele frequency higher than 0.05 in five populations (Table S4). Therefore, locus *EF‐28* was excluded from all subsequent analyses. None of the retained 19 loci showed a consistent deviation from HWE in the populations or different life stages, and all locus pairs were in equilibrium linkage.

The population of SKY village had the highest genetic variation with *A*_R_, *H*_O,_ and *H*_E_ values of 3.3219, 0.5467, and 0.5330, respectively, and the populations of DH Mountain had the highest *A*_P_ value of 0.3089. The population of WYG village had the lowest genetic variation with *A*_R_, *A*_P_ and *H*_E_ values of 2.8472, 0.0268, and 0.4007, respectively, and the population of DH Mountain had the lowest *H*_O_ value of 0.4631 (Table 1). One‐sided Wilcoxon test indicated that all genetic variations (*A*_R_, *A*_P_, *H*_O,_ and *H*_E_) were not significantly different between populations except for WYG and SKY villages in *H*_E_ value and WYG village and DH Mountain in *A*_P _value (Table S5). The inbreeding coefficients (*f*) in the populations ranged from −0.1647 to −0.0134, and almost all the populations showed a significant deviation from zero due to heterozygosity excess except LT village and DH Mountain (Table 1).

Analysis of the different life stages revealed the populations of LT villages had higher genetic variation of all *A*_R_, *A*_P_, *H*_O,_ and *H*_E_ values in older generations (adult and juvenile) than in seedlings, but the populations of WYG, ZL, and SKY villages had the highest in seedlings, the youngest generation (Table 1). However, for each *Feng Shui* population, the differences in genetic variation among generations were not significant (Table S6). All *f* values in the life stages of the populations were smaller than zero, but not all of them significant deviation from zero (Table 1).

**Figure 3:**
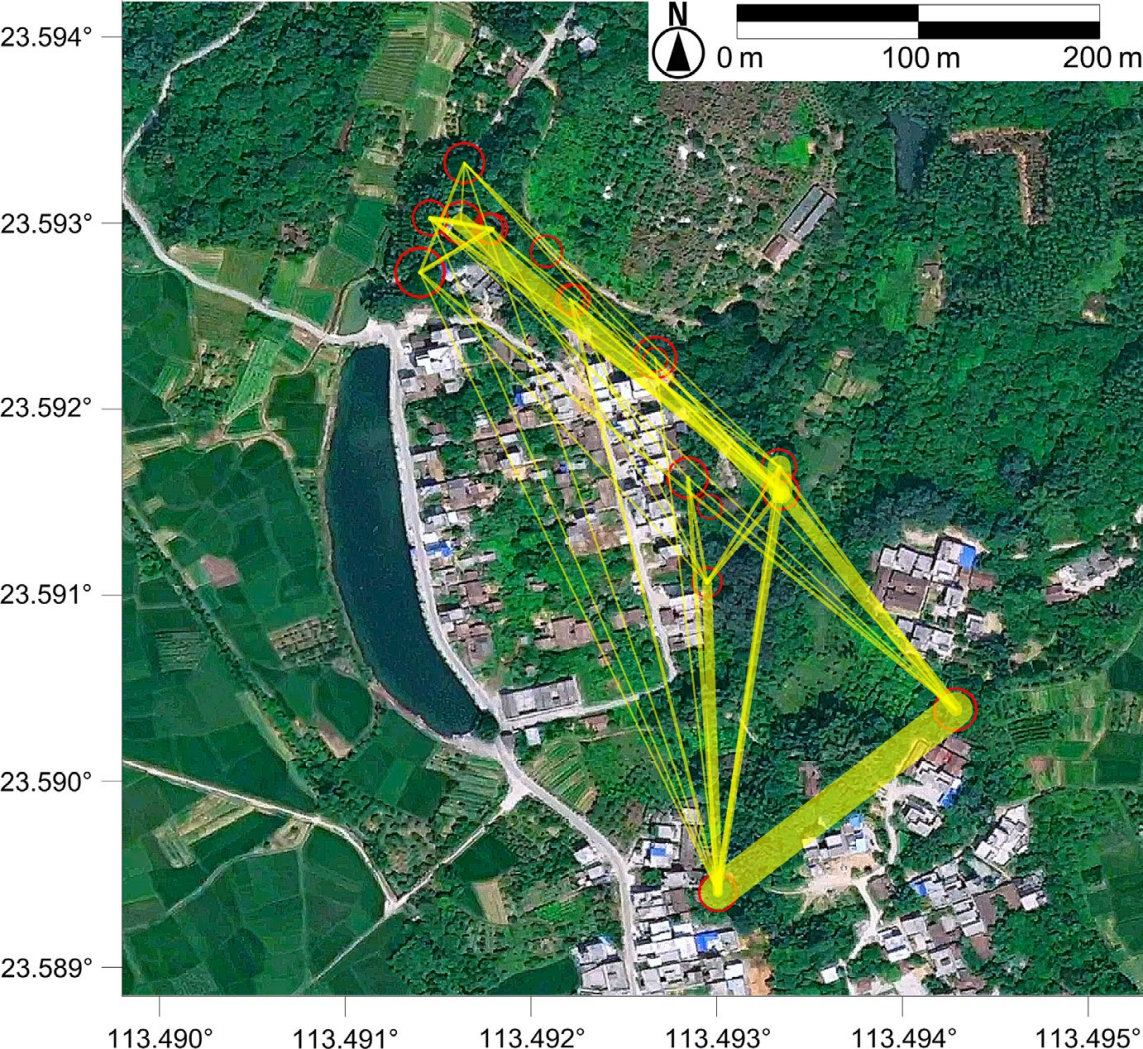
Picture showing pollen flows among adult individuals. The width of the lines between two adult trees corresponds to the amount of pollen flow

The combined nonexclusion probabilities across the 19 microsatellite loci for the first parent, second parent, and parent pairs were 0.0407078, 0.00204951, and 0.00003035, respectively. The combined nonexclusion probability of identity was 1.654 × 10^–10^. The probability of cryptic gene flow was 0.0005462. All these results indicated the microsatellites used here had high power of exclusion and were optimal for parentage and paternity analyses. Then, parentage analysis successfully identified the parents for 206 (80.16%) of the 257 seedlings in the population of TB village at a 95% confidence level. We assigned a mother trees for 49 of the 51 remaining seedlings according to their geographic position. Paternity analysis then successfully identified the pollen donating trees for 12 of the 49 at the 95% confidence level. Therefore, the parents of 218 seedlings were identified. The distances of the parent–offspring pairs detected ranged from 2.275 to 457.959 m with mean of 160.085 m (Figure 3), excluding 30 self‐fertilization cases. The self‐fertilization rate was 0.117 (30/257). The actual pollen immigration rate was 0.144 (37/257), and the effective pollination neighboring area (*A*_ep_) was 11.241 ha.

For the population of TB village, the neighborhood model showed the self‐fertilization rate was 0.122 (*SE* 0.021), the pollen immigration rate 0.134 (*SE* 0.023), the mean pollen dispersal distance 220.910 m (*SE* 24.836), the seed immigration rate 0.010 (*SE* 0.007), and the mean seed dispersal distance 4.666 m (*SE* 4.724).

**Figure 4:**
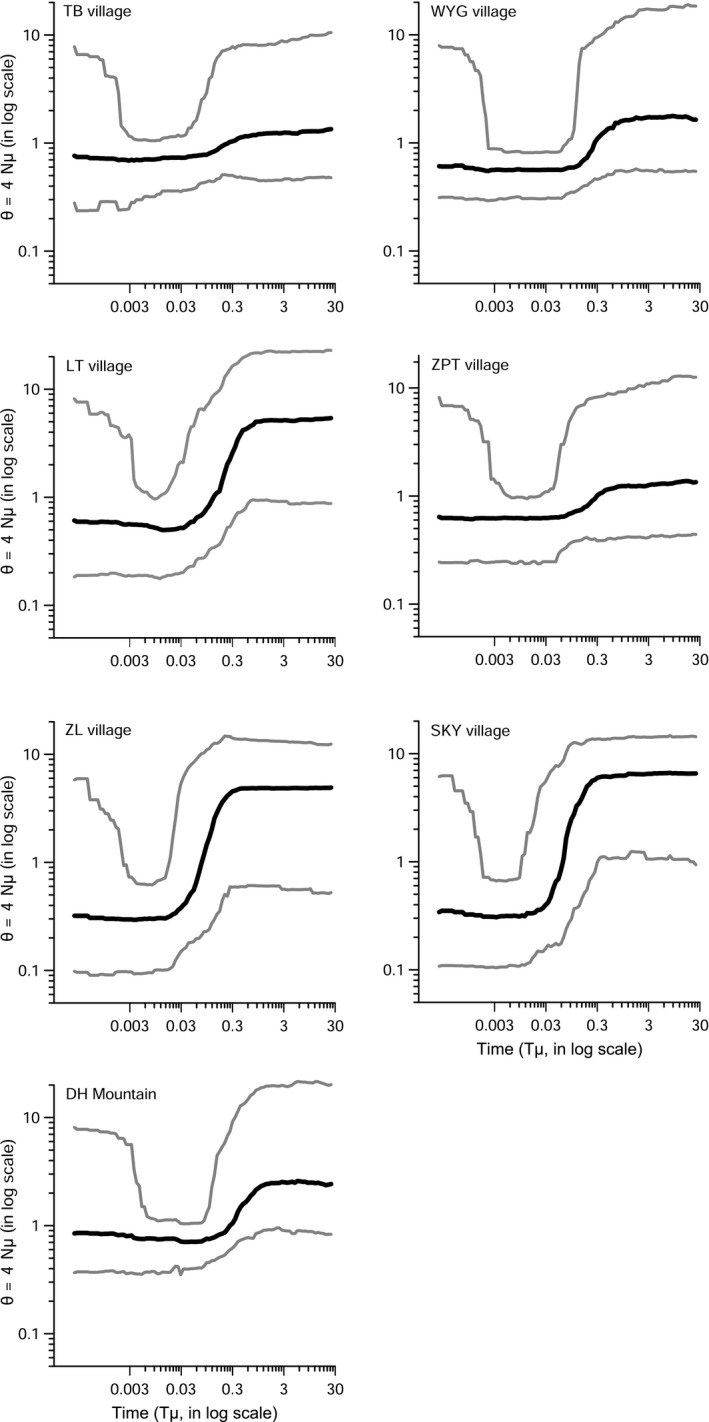
Bayesian skyline plots showing the demographic history of *Erythrophleum fordii* for different populations. The black line shows the median estimates of historical population size (*θ*), and the gray lines around the median estimates show the 95% highest posterior density estimates of the historical population size. Note that the most recent time is on the left of the *x*‐axis. *N* is effective population size, *μ* is the mutation rate per generation, and *T* is the time in generations

With the exceptions of the populations of WYG and DH Mountain using the heterozygosity‐excess method, both heterozygosity‐excess and M‐ratio methods indicated that all the *E*. *fordii* populations analyzed were likely to have experienced a past bottleneck at *p* < .05 (Table 2). Furthermore, DIYABCskylineplot detected a clear demographic decline in recent history in all the populations tested (Figure 4).

**Figure 5:**
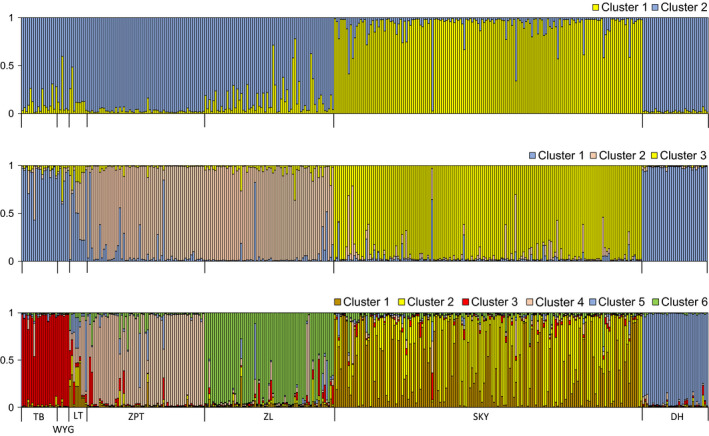
Membership probability results of the STRUCTURE analyses based on adults for seven *Erythrophleum fordii* populations. STRUCTURE shows genetic group *K* = 2, 3, and 6. In the plot each vertical bar represents one individual and the colors in the bar show the assignment probability to the genetic clusters

The mean Log‐likelihood values in STRUCTURE analysis indicated *K* = 6 was the “optimal” genetic clusters (Figure S5) because at *K* = 6 the Log‐likelihood values began to reach “more‐or‐less plateaus” according to the STRUCTURE manual. The Δ*K* showed two obvious peaks with the highest at *K* = 2 and the second highest at *K* = 6. However, because Δ*K* value was smaller at *K* = 6 than at *K* = 3, we then illustrated these three *K* results (Figure 5). At *K* = 2, the population of SKY village formed a distinctive cluster separated from the other populations. At *K* = 3, the population of SKY village remained distinctive, and the populations of ZPT and ZL villages were separated from the rest four populations. At *K* = 6, all the populations formed their own distinctive clusters except the populations of TB and WYG villages, and the population of SKY village showed clear genetic admixtures.

## 4 DISCUSSION

### 4.1 Genetic diversity

Among approximately 15 species in the *Erythrophleum* genus, which is mainly found in Africa, only *E*. *fordii* is found in China. Since it is an endangered species, it is valuable to compare its level of genetic diversity to that of its congeners (Cole, 2003; Gitzendanner & Soltis, 2000). However, few studies have reported the genetic diversity of *Erythrophleum* species. The only study was by Duminil et al. (2016) who examined the genetic diversity of *E*. *suaveolens* in Africa to estimate the impact of logging on population dynamics. Similar to our study, this study also investigated the genetic variation in different life stages of *E*. *suaveolens*. It reported *H*_O_ and *H*_E_ values ranging from 0.461 to 0.531 and 0.535 to 0.658, respectively. Therefore, the genetic variation of *E*. *fordii* in the different life stages in our *Feng Shui* populations had lower *H*_E_ values, which ranged from 0.4007 to 0.5379 (Table 1) than *E*. *suaveolens*, but comparable *H*_O_ values to *E*. *suaveolens*.

We previously investigated the genetic diversity of *E*. *fordii* in one well‐preserved population using nine microsatellites in the DH Mountain, a nature reserve. Although our previous study reported higher genetic diversity in the DH Mountain (a *H*_O_ of 0.606 and *H*_E_ of 0.586, Zhu et al., 2013) than all the *Feng Shui* populations we investigated here, our present study using a new set of microsatellites indicated this was not the case (Table 1). In fact, the genetic diversity of the population of DH Mountain was only significantly higher than that of the population of WYG village in *A*_P_ value (Table S5). This implies, on one hand, that *E*. *fordii* in *Feng Shui* woodlands are not deprived of genetic diversity; on the other hand, that at population level, the genetic diversities within populations measured using different sets of markers should be compared with caution.

We observed that among all the populations, SKY harbored the highest genetic diversity but not the highest number of private alleles (Table 1). A possible reason for this may be that this population was artificially established with high numbers from many different resources which already somewhat depleted in alleles. Compared to the other populations, substantial admixture revealed by STRCTURE analysis (*K* = 6) confirmed the admixture. However, to our surprise, these sources could not be from nearby populations we sampled. In ancient times, without efficient transportation tools, the troop stationed in SKY that planted the trees might not have been able to introduce *E*. *fordii* individuals from places far away. Since there are no the other *E*. *fordii* populations geographically closer to the population of SKY village than the populations we sampled, a possible explanation is that the source population(s) of SKY village have been extinct under urbanization. Nevertheless, this hypothesis needs further studies with more extensive sampling than ours in the future.

For the other genetic diversity parameter, inbreeding coefficients (*f* values), Duminil et al. (2016) observed high *f* values in young life stages (seed and/or seedling) of *E*. *suaveolens*, regardless of the population densities (ranging from 0.7 to 1.72 individuals per ha with DBH >30 cm), which indicates that their populations had high selfing and inbreeding rates. Our *Feng Shui* populations of *E*. *fordii* did not seem to have suffered from inbreeding because all life stages had near zero *f* values (Table 1), which indicates random mating. Such random mating also existed in the well‐preserved population of DH Mountain whose *f* value was 0.0082 (Table 1). This is consistent with our previous data with *f* values of −0.058 to 0.022 in different age classes in the population of DH Mountain (Zhu et al., 2013). Since *E*. *fordii* and *E*. *suaveolens* live in two geographically distant continents and may have undergone very different evolutionary processes, including pollen mutualisms, the reason behind the difference in their mating patterns, revealed by their *f* values, may be difficult to interpret currently. In fact the high *f* value in the early life stage of *E*. *suaveolens* was uninterpretable by Duminil et al. (2016).

### 4.2 Pollination and pollen flow

Our parentage analysis for the TB village population (Figure 3) further supports the conclusion of random mating in *E*. *fordii*. Although this figure shows that mating events seem to be biased to a few individuals, our field investigation indicated this may not be true in nature. TB village is an environment with lots of human interference, and for most of the mother trees, their existing environment is clearly unsuitable for seedling survival (Figure S4). Below these trees, few or no recruits were found (Figure 2). Since our parentage analysis was based on seedlings collected in the field, these few mother trees would display higher mating events than most of the others, resulting in skewed mating patterns in the population (Figure 3). Given this, we believe that the pollination network required random mating in *E*. *fordii* populations, at least in the TB village population, was not destroyed by urbanization.

According to the review by Senapathi, Goddard, Kunin, and Baldock (2017), pollinator abundance and composition in urbanized areas are not always inferior to less disturbed areas. The flowers of *E*. *fordii* can attract a diverse range of pollinators (Zhu et al., 2013). Therefore, the positive effects of the pollinators may compensate for the negative effects of urbanization to maintain a stable random mating system in *Feng Shui* populations of *E*. *fordii*, even if the pollinator fauna or composition among disturbed and less disturbed populations is different. For example, by comparing the genetic variations of *Syagrus romanzoffiana* between continuously protected and nearby fragmented forests in South America, Giombini et al. (2017) did not find that pollen donors were affected in the latter. However, further pollinator fauna observations are needed to better interpret our results.

In this study, we found pollen immigration rate (*mp*) of 0.144 and 0.122, and mean pollen dispersal distance (*dp*) of 160.085 and 220.910 m based on parentage assignments and neighborhood model, respectively, in the population of TB village. Effective pollination neighborhood (*A*_ep_) for *E*. *fordii* was 11.24 ha between seed trees, equivalent to a circle with a radius of 189.21 m around a seed tree. Hence, considering the very limited seed immigration rate of 0.010 and dispersal distance of 4.666 m we observed, long‐distance gene flow in *E*. *fordii* is primarily by pollen dispersal. As both mp and dp are directly related to the sizes and degrees of isolation of areas, they should vary among species and populations within species with similar pollination insects (Braga & Collevatti, 2011; Manoel et al., 2012; Monthe, Hardy, Doucet, Loo, & Duminil, 2016; Noreen et al., 2016; Sebbenn et al., 2011; Tambarussi, Boshier, Vencovsky, Freitas, & Sebbenn, 2015). For example, for a Neotropical tree *Copaifera langsdorffii* in a highly isolated and fragmented forest fragment (4.8 ha), Manoel et al. (2012) found its *mp* and *dp* values were 0.08 and 66 m, respectively; while for a tropical tree *Entandrophragma cylindricum* in a relatively large and continuous forest, Monthe et al. (2016) found its *mp* and *dp* values were 0.32–0.40 and 506–540 m, respectively. Therefore, we consider the pollen flow in the population of TB village moderate. Because pollination insects, such as bees, could carry pollen to very long distances (Braga & Collevatti, 2011; Dick, Etchelecu, & Austerlitz, 2003; Manoel et al., 2012; Noreen et al., 2016; Sebbenn et al., 2011; Tambarussi et al., 2015), it is possible to find larger mp and dp in larger and more continuous populations of *E*. *fordii* than those in our study, based on pollen‐mediated gene dispersal capacities of *E*. *fordii*. Because our study here is mainly focused on local pollination dynamics, we provide no indication of the potential pollen sources for immigrated pollens. Continued research including more surrounding populations on characterizing within and among population pollen flow patterns is a priority in this system in the future.

### 4.3 Demographic history

Our data indicate that all of our populations clearly suffered from bottlenecks. It is possible that such bottlenecks might have been caused by recent (approximately 100 years ago) wood demands due to long periods of war and poverty. However, the results from skyline plots (Figure 4) indicate that dramatic declines in population size could be dated back 1,000 years ago. The skyline plot is based on an ABC (Approximate Bayesian computation) framework and is mostly likely to detect large events which influenced population size dramatically, whereas demographic changes of small magnitude and close to the present are the hardest to detect (Navascués et al., 2017).

Among the populations, if we consider the point at 0.3 on the *x*‐axis (measuring the time scale) when the skyline plot results suggest the most recent ancestral decline with a high magnitude started (Figure 4), 20–25 years per generation for *E*. *fordii* (Tang et al., 2015), and 5 × 10^–4^ mutations per locus per generation for all microsatellite loci (Melo, Freitas, Bacon, & Collevatti, 2018), we could convert the time scale to 12,000–15,000 years, which is when the last ice age started to end and humans entered the New Stone Age. However, an accurate prediction of a major historical scenario is still challenging and influenced by many factors (Navascués et al., 2017). Therefore, the above time period related to the demographic declines in *E*. *fordii* populations should be interpreted with caution, and earlier or later historical events might also have played a role. According to Meng, Wang, Hu, Zhang, and Lai (2017), southern China underwent a short period of cold weather about 8,500 years ago, which had a big impact on the tropical rainforest trees. Thus, as a thermophilic species, *E*. *fordii* populations might have shrunk since then.

How the demographic decline in *E*. *fordii* was caused by human activities in the past is unclear. However, due to its high quality timber, *E*. *fordii* had been in high demand in historical markets and was recorded as one of the “four famous woods” in the Ming dynasty (established in 1368 A.D.) in China (Liang, 2014). According to historical records, the early use of *E*. *fordii* can be dated back to the Qin dynasty (established 221 B.C.), and more records of its use have been found since the Song dynasty (established in 960 A.D.) (Zhou, 2007). One famous use of *E*. *fordii* in China was to build Zhenwu Pavilion, which was completely built using approximately 3,000 pieces of *E*. *fordii* timber in 1573 A.D. In Vietnam, *E*. *fordii* has also been reported to be used in many historical buildings (Nguyen et al., 2018). Nevertheless, the high wood demand might have prompted people historically to establish artificial plantations such as the *E*. *fordii* population in SKY village, which would alleviate the shortage of naturally grown *E*. *fordii* timber. This could be why *E*. *fordii* is frequently found in *Feng Shui* villages where human activities are intensive. In the future, the use of large‐scale sampling and whole genome information for *E*. *fordii* will more clearly reveal its historical evolutionary dynamics.

### 4.4 Conservation implication

Our results show that despite moderate gene flow via pollen, the limited seed dispersal distance may result in significant relatedness among *E*. *fordii* individuals at short distances. Furthermore, they reveal clearly disproportionate contributions of adults to the recruit landscape due to absence of suitable recruitment environment under some adults, which could remain across reproductive cycles if present unfavorable conditions for seedling establishment continue. Together with substantial self‐fertilization which is often associated with isolated and fragmented small populations (Cheptou, Hargreaves, Bonte, & Jacquemyn, 2017), all these factors may increase the rates of mating among relatives, producing negative fitness effects in future generations, and slowing down adaptation in the face of climate change.

Seed collection for ex‐situ conservation of *E*. *fordii* should include *Feng Shui* woodlands as high genetic diversity harbored in most them and contain many trees as many trees as possible. The mean pollen dispersal distance suggests that seed trees must be separated by at least 220 m. In addition, since the gene pool of the population of SKY village is different from the others, seeds collected from it should be separated and only mixed with those from other populations after being sure no outbreeding depression effects happening.

## 5 CONCLUSION

*Feng Shui* woodlands, as part of CPFs, are valuable supplements for urban forests in southern China, especially with the present policy aiming to build national forest cities, because they are more natural and have higher species diversities than modern artificial green lands (Ye et al., 2013). Furthermore, they can provide a source for rural afforestation and play a key role in ecological networks. Globally, CPFs are distinctive elements of worldwide vegetations, and they naturally and seminaturally distribute in and around urban areas (Avtzis et al., 2018; Bossart & Antwi, 2016; Hu et al., 2011; Lee et al., 2019). Similar to *Feng Shui* woodlands in China, most of the plant species in CPFs are not considered being threatened in the near future. However, monitoring their gene pools is essential to prevent genetic erosion caused by anthropogenic effects. Therefore, an extension of this study to other species with different life stages and different landscape configures in CPFs would be recommended to know their gene flow and how such flow determines the microevolutionary changes in them.

Overall, our results suggest that *E*. *fordii* may have suffered serious demographic declines before large‐scale human settlements in southern China and has not recovered at the present time due to consistently high demands for its high quality wood. However, parentage analysis indicated that its pollen‐mediated gene flows were not severely affected within the disturbed suburban areas, and genetic diversity was stably maintained across different generations. A previous simulation study of *E*. *fordii* also indicated that its longevity with iteroparity provided the potential to maintain genetic diversity in small isolated populations (Zhu et al., 2013), and our present study supports this conclusion. However, for most of the *Feng Shui* woodlands, the major threat to the long‐term adaptation and evolution of *E*. *fordii* is the lack of suitable regeneration habitats. The maintenance of large and continuous populations to guarantee high gene flow is also required for long‐term species sustainability.

## CONFLICT OF INTEREST

The authors declare no competing interests.

## AUTHOR CONTRIBUTIONS

WZF conceived and designed the project and carried out the laboratory procedures and data analyses. WZF and LHL carried out the field collections. All authors contributed to writing the manuscript.

## Data Availability

The RAD sequences data are available in the NCBI SRA database with accession numbers SRX5010692 and SRX5010693. The microsatellites are available in the NCBI database with accession numbers shown in Table S2. Sample information and full microsatellite dataset are deposited in ResearchGate (https://www.researchgate.net/publication/333131062_Table_S7, https://doi.org/10.13140/RG.2.2.17309.56807).

## ENDNOTE

1
www.iucnredlist.org

## References

[ece35513-bib-0001] Archer, F. I. , Adams, P. E. , & Schneiders, B. B. (2017). Stratag: An r package for manipulating, summarizing and analysing population genetic data. Molecular Ecology Resources, 17, 5–11. 10.1111/1755-0998.12559 27327208

[ece35513-bib-0002] Avtzis, D. N. , Stara, K. , Sgardeli, V. , Betsis, A. , Diamandis, S. , Healey, J. R. , … Halley, J. M. (2018). Quantifying the conservation value of Sacred Natural Sites. Biological Conservation, 222, 95–103. 10.1016/j.biocon.2018.03.035

[ece35513-bib-0003] Baird, N. A. , Etter, P. D. , Atwood, T. S. , Currey, M. C. , Shiver, A. L. , Lewis, Z. A. , … Johnson, E. A. (2008). Rapid SNP discovery and genetic mapping using sequenced RAD markers. PLoS ONE, 3(10), e3376 10.1371/journal.pone.0003376 18852878 PMC2557064

[ece35513-bib-0004] Bartlewicz, J. , Vandepitte, K. , Jacquemyn, H. , & Honnay, O. (2015). Population genetic diversity of the clonal self‐incompatible herbaceous plant *Linaria vulgaris* along an urbanization gradient. Biological Journal of the Linnean Society, 116, 603–613. 10.1111/bij.12602

[ece35513-bib-0005] Bossart, J. L. , & Antwi, J. B. (2016). Limited erosion of genetic and species diversity from small forest patches: Sacred forest groves in an Afrotropical biodiversity hotspot have high conservation value for butterflies. Biological Conservation, 198, 122–134. 10.1016/j.biocon.2016.03.029

[ece35513-bib-0006] Braga, A. C. , & Collevatti, R. G. (2011). Temporal variation in pollen dispersal and breeding structure in a bee‐pollinated Neotropical tree. Heredity, 106, 911–919. 10.1038/hdy.2010.134 20978531 PMC3186249

[ece35513-bib-0007] Burczyk, J. , Adams, W. T. , Birkes, D. S. , & Chybicki, I. J. (2006). Using genetic markers to directly estimate gene flow and reproductive success parameters in plants on the basis of naturally regenerated seedlings. Genetics, 173, 363–372. 10.1534/genetics.105.046805 16489237 PMC1461435

[ece35513-bib-0008] Cheptou, P.‐O. , Hargreaves, A. L. , Bonte, D. , & Jacquemyn, H. (2017). Adaptation to fragmentation: Evolutionary dynamics driven by human influences. Philosophical Transactions of the Royal Society B: Biological Sciences, 372, 20160037 10.1098/rstb.2016.0037 PMC5182433 27920382

[ece35513-bib-0009] Chong, Z. , Ruan, J. , & Wu, C. I. (2012). Rainbow: An integrated tool for efficient clustering and assembling RAD‐seq reads. Bioinformatics, 28, 2732–2737. 10.1093/bioinformatics/bts482 22942077

[ece35513-bib-0010] Chybicki, I. J. , & Burczyk, J. (2009). Simultaneous estimation of null alleles and inbreeding coefficients. Journal of Heredity, 100, 106–113. 10.1093/jhered/esn088 18936113

[ece35513-bib-0011] Chybicki, I. J. , & Burczyk, J. (2010). NM+: Software implementing parentage‐based models for estimating gene dispersal and mating patterns in plants. Molecular Ecology Resources, 10, 1071–1075. 10.1111/j.1755-0998.2010.02849.x 21565118

[ece35513-bib-0012] Coggins, C. (2003). The tiger and the pangolin: Nature, culture, and conservation in China. Honolulu, HI: University of Hawaii Press.

[ece35513-bib-0013] Cole, C. T. (2003). Genetic variation in rare and common plants. Annual Review of Ecology, Evolution, and Systematics, 34, 213–237. 10.1146/annurev.ecolsys.34.030102.151717

[ece35513-bib-0014] Cornuet, J. M. , & Luikart, G. (1996). Description and power analysis of two tests for detecting recent population bottlenecks from allele frequency data. Genetics, 144, 2001–2014. 8978083 10.1093/genetics/144.4.2001 PMC1207747

[ece35513-bib-0015] Dick, C. W. , Etchelecu, G. , & Austerlitz, F. (2003). Pollen dispersal of tropical trees (*Dinizia excelsa*: Fabaceae) by native insects and African honeybees in pristine and fragmented Amazonian rainforest. Molecular Ecology, 12, 753–764. 10.1046/j.1365-294X.2003.01760.x 12675830

[ece35513-bib-0016] Dow, B. D. , & Ashley, M. V. (1996). Microsatellite analysis of seed dispersal and parentage of sampling in bur oak, *Quercus macrocarpa* . Molecular Ecology, 5, 615–627. 10.1111/j.1365-294X.1996.tb00357.x

[ece35513-bib-0017] Dubois, J. , & Cheptou, P.‐O. (2017). Effects of fragmentation on plant adaptation to urban environments. Philosophical Transactions of the Royal Society B: Biological Sciences, 372, 20160038 10.1098/rstb.2016.0038 PMC5182434 27920383

[ece35513-bib-0018] Duminil, J. , Daïnou, K. , Kaviriri, D. K. , Gillet, P. , Loo, J. , Doucet, J.‐L. , & Hardy, O. J. (2016). Relationships between population density, fine‐scale genetic structure, mating system and pollen dispersal in a timber tree from African rainforests. Heredity, 116, 295–303. 10.1038/hdy.2015.101 26696137 PMC4806568

[ece35513-bib-0019] Evanno, G. , Regnaut, S. , & Goudet, J. (2005). Detecting the number of clusters of individuals using the software STRUCTURE: A simulation study. Molecular Ecology, 14, 2611–2620. 10.1111/j.1365-294X.2005.02553.x 15969739

[ece35513-bib-0020] Faircloth, B. C. (2008). MSATCOMMANDER: Detection of microsatellite repeat arrays and automated, locus‐specific primer design. Molecular Ecology Resources, 8, 92–94. 10.1111/j.1471-8286.2007.01884.x 21585724

[ece35513-bib-0021] Flint‐Garcia, S. A. , Thornsberry, J. M. , & Buckler, E. S. (2003). Structure of linkage disequilibrium in plants. Annual Review of Plant Biology, 54, 357–374. 10.1146/annurev.arplant.54.031902.134907 14502995

[ece35513-bib-0022] Garza, J. C. , & Williamson, E. G. (2001). Detection of reduction in population size using data from microsatellite loci. Molecular Ecology, 10, 305–318. 10.1046/j.1365-294X.2001.01190.x 11298947

[ece35513-bib-0023] Ge, Y. J. , Liu, Y. J. , Shen, A. H. , & Lin, X. C. (2015). Fengshui forests conserve genetic diversity: A case study of *Phoebe bournei* (Hemsl.) Yang in southern China. Genetics and Molecular Research, 14, 1986–1993. 10.4238/2015.March.20.8 25867344

[ece35513-bib-0024] Giombini, M. I. , Bravo, S. P. , Sica, Y. V. , & Tosto, D. S. (2017). Early genetic consequences of defaunation in a large‐seeded vertebrate‐dispersed palm (*Syagrus romanzoffiana*). Heredity, 118, 568–577. 10.5061/dryad.s0vf6 28121308 PMC5436022

[ece35513-bib-0025] Gitzendanner, M. A. , & Soltis, P. S. (2000). Patterns of genetic variation in rare and widespread plant congeners. American Journal of Botany, 87, 783–792. 10.2307/2656886 10860909

[ece35513-bib-0026] Guan, C. Y. (2002). Investigation on geomantic forest in ancient China. Agricultural Archaeology, 3, 239–243. [In Chinese with English abstract].

[ece35513-bib-0027] Hermansen, T. D. , Roberts, D. G. , Toben, M. , Minchinton, T. E. , & Ayre, D. J. (2015). Small urban stands of the mangrove *Avicennia marina* are genetically diverse but experience elevated inbreeding. Estuaries and Coasts, 38, 1898–1907. 10.1007/s12237-015-9955-1

[ece35513-bib-0028] Holm, S. (1979). A simple sequentially rejective multiple test procedure. Scandinavian Journal of Statistics, 6, 65–70.

[ece35513-bib-0029] Honnay, O. , & Bossuyt, B. (2005). Prolonged clonal growth: Escape route or route to extinction? Oikos, 108, 427–432. 10.1111/j.0030-1299.2005.13569.x

[ece35513-bib-0030] Hu, L. , Li, Z. , Liao, W.‐B. , & Fan, Q. (2011). Values of village fengshui forest patches in biodiversity conservation in the Pearl River Delta, China. Biological Conservation, 144, 1553–1559. 10.1016/j.biocon.2011.01.023

[ece35513-bib-0031] Huang, X. , & Madan, A. (1999). CAP3: A DNA sequence assembly program. Genome Research, 9, 868–877. 10.1101/gr.9.9.868 10508846 PMC310812

[ece35513-bib-0032] Jakobsson, M. , & Rosenberg, N. A. (2007). CLUMPP: A cluster matching and permutation program for dealing with label switching and multimodality in analysis of population structure. Bioinformatics, 23, 1801–1806. 10.1093/bioinformatics/btm233 17485429

[ece35513-bib-0033] Johnson, M. T. J. , & Munshi‐South, J. (2017). Evolution of life in urban environments. Science, 358, eaam8327 10.1126/science.aam8327 29097520

[ece35513-bib-0034] Johnson, M. T. J. , Thompson, K. A. , & Saini, H. S. (2015). Plant evolution in the urban jungle. American Journal of Botany, 102, 1951–1953. 10.3732/ajb.1500386 26620097

[ece35513-bib-0035] Kalinowski, S. T. , Taper, M. L. , & Marshall, T. C. (2007). Revising how the computer program CERVUS accommodates genotyping error increases success in paternity assignment. Molecular Ecology, 16, 1099–1106. 10.1111/j.1365-294X.2007.03089.x 17305863

[ece35513-bib-0036] Lee, J. H. , Hong, S. H. , & Kim, D. W. (2019). The effectiveness of the conservation of Korean village groves (Maeulsoop) based on an assessment of tree ages. Urban Forestry & Urban Greening, 39, 26–34. 10.1016/j.ufug.2019.02.006

[ece35513-bib-0037] Levin, D. A. (1988). The paternity pools of plants. The American Naturalist, 132, 309–317. 10.1086/284854

[ece35513-bib-0038] Liang, R.‐L. (2014). *Erythrophleum fordii*: Chinese redwood. Forestry of Guangxi, 10, 28–29. [In Chinese with English abstract].

[ece35513-bib-0039] Lowe, A. J. , Cavers, S. , Boshier, D. , Breed, M. F. , & Hollingsworth, P. M. (2015). The resilience of forest fragmentation genetics ‐no longer a paradox‐ we were just looking in the wrong place. Heredity, 115, 97–99. 10.1038/hdy.2015.40 26176685 PMC4815445

[ece35513-bib-0040] Manoel, R. O. , Alves, P. F. , Dourado, C. L. , Gaino, A. P. S. C. , Freitas, M. L. M. , Moraes, M. L. T. , & Sebbenn, A. M. (2012). Contemporary pollen flow, mating patterns and effective population size inferred from paternity analysis in a small fragmented population of the Neotropical tree *Copaifera langsdorffii* Desf. (Leguminosae‐Caesalpinioideae). Conservation Genetics, 13, 613–623. 10.1007/s10592-011-0311-0

[ece35513-bib-0041] Martinez, J. J. I. , & Amar, Z. (2014). The preservation value of a tiny sacred forest of the oak *Quercus calliprinos* and the impact of livestock presence. Journal of Insect Conservation, 18, 657–665. 10.1007/s10841-014-9672-2

[ece35513-bib-0042] McDonald, M. J. , Rice, D. P. , & Desai, M. M. (2016). Sex speeds adaptation by altering the dynamics of molecular evolution. Nature, 531, 233–236. 10.1038/nature17143 26909573 PMC4855304

[ece35513-bib-0043] Melo, W. A. , Freitas, C. G. , Bacon, C. D. , & Collevatti, R. G. (2018). The road to evolutionary success: Insights from the demographic history of an Amazonian palm. Heredity, 121, 183–195. 10.1038/s41437-018-0074-1 29588509 PMC6039527

[ece35513-bib-0044] Meng, Y. , Wang, W. , Hu, J. , Zhang, J. , & Lai, Y. (2017). Vegetation and climate changes over the last 30 000 years on the Leizhou Peninsula, southern China, inferred from the pollen record of Huguangyan Maar Lake. Boreas, 46, 525–540. 10.1111/bor.12229

[ece35513-bib-0045] Monthe, F. K. , Hardy, O. J. , Doucet, J. L. , Loo, J. , & Duminil, J. (2016). Extensive seed and pollen dispersal and assortative mating in the rain forest tree *Entandrophragma cylindricum* (Meliaceae) inferred from indirect and direct analyses. Molecular Ecology, 26, 5279–5291. 10.1111/mec.14241 28734064

[ece35513-bib-0046] Nagamitsu, T. , Kikuchi, S. , Hotta, M. , Kenta, T. , & Hiura, T. (2014). Effects of population size, forest fragmentation, and urbanization on seed production and gene flow in an endangered maple (*Acer miyabei*). The American Midland Naturalist Journal, 172, 303–316. 10.1674/0003-0031-172.2.303

[ece35513-bib-0047] Navascués, M. , Leblois, R. , & Burgarella, C. (2017). Demographic inference through approximate‐Bayesian‐computation skyline plots. PeerJ, 5, e3530 10.7717/peerj.3530 28729953 PMC5518730

[ece35513-bib-0048] Nguyen, T. D. , Nishimura, H. , Imai, T. , Watanabe, T. , Kohdzuma, Y. , & Sugiyama, J. (2018). Natural durability of the culturally and historically important timber: *Erythrophleum fordii* wood against white‐rot fungi. Journal of Wood Science, 64, 301–310. 10.1007/s10086-018-1704-1

[ece35513-bib-0049] Noreen, A. , Niissalo, M. A. , Lum, S. K. Y. , & Webb, E. L. (2016). Persistence of long‐distance, insect‐mediated pollen movement for a tropical canopy tree species in remnant forest patches in an urban landscape. Heredity, 117, 472–480. 10.1038/hdy.2016.64 27703155 PMC5117842

[ece35513-bib-0050] Owusu, S. A. , Schlarbaum, S. E. , Carlson, J. E. , & Cailing, O. (2016). Pollen gene flow and molecular identification of full‐sib families in small and isolated population fragments of *Gleditsia triacanthos* L. Botany‐Botanique, 94, 523–532. 10.1139/cjb-2015-0244

[ece35513-bib-0051] Peakall, R. , & Smouse, P. (2012). GenAlEx 6.5: Genetic analysis in Excel. Population genetic software for teaching and research ‐ An update. Bioinformatics, 28, 2537–2539. 10.1093/bioinformatics/bts460 22820204 PMC3463245

[ece35513-bib-0052] Pritchard, J. K. , Stephens, M. , & Donnelly, P. (2000). Inference of population structure using multilocus genotype data. Genetics, 155, 945–959. 10835412 10.1093/genetics/155.2.945 PMC1461096

[ece35513-bib-0053] Rosas, F. , Quesada, M. , Lobo, J. A. , & Sork, V. L. (2011). Effects of habitat fragmentation on pollen flow and genetic diversity of the endangered tropical tree *Swietenia humilis* (Meliaceae). Biological Conservation, 144, 3082–3088. 10.1016/j.biocon.2011.10.003

[ece35513-bib-0054] Rousset, F. (2008). GENEPOP'007: A complete re‐implementation of the genepop software for Windows and Linux. Molecular Ecology Resources, 8, 103–106. 10.1111/j.1471-8286.2007.01931.x 21585727

[ece35513-bib-0055] Schwarcz, K. D. , Silvestre, E. D. A. , de Campos, J. B. , Sujii, P. S. , Grando, C. , Macrini, C. M. T. , … Zucchi, M. I. (2018). Shelter from the storm: Restored populations of the neotropical tree *Myroxylon peruiferum* are as genetically diverse as those from conserved remnants. Forest Ecology and Management, 410, 95–103. 10.1016/j.foreco.2017.12.037

[ece35513-bib-0056] Sebbenn, A. M. , Carvalho, A. C. M. , Freitas, M. L. M. , Moraes, S. M. B. , Gaino, A. P. S. C. , da Silva, J. M. , … Moraes, M. L. T. (2011). Low levels of realized seed and pollen gene flow and strong spatial genetic structure in a small, isolated and fragmented population of the tropical tree *Copaifera langsdorffii* Desf. Heredity, 106, 134–145. 10.1038/s41437-019-0231-1 20372183 PMC3183851

[ece35513-bib-0057] Senapathi, D. , Goddard, M. A. , Kunin, W. E. , & Baldock, K. C. R. (2017). Landscape impacts on pollinator communities in temperate systems: Evidence and knowledge gaps. Functional Ecology, 31, 26–37. 10.1111/1365-2435.12809

[ece35513-bib-0058] Szpiech, Z. A. , Jakobsson, M. , & Rosenberg, N. A. (2008). ADZE: A rarefaction approach for counting alleles private to combinations of populations. Bioinformatics, 24, 2498–2504. 10.1093/bioinformatics/btn478 18779233 PMC2732282

[ece35513-bib-0059] Tambarussi, E. V. , Boshier, D. , Vencovsky, R. , Freitas, M. L. M. , & Sebbenn, A. M. (2015). Paternity analysis reveals significant isolation and near neighbor pollen dispersal in small *Cariniana legalis* Mart. Kuntze populations in the Brazilian Atlantic Forest. Ecology and Evolution, 5, 5588–5600. 10.1002/ece3.1816 27069608 PMC4813111

[ece35513-bib-0060] Tang, J.‐X. , Ma, J. , Jia, H.‐Y. , Zeng, Y. , Lei, Y.‐C. , & Cai, D.‐X. (2015). Study on the growth law of a rare and endangered tree species of Erythrophleum fordii in south subtropical Area of China. Journal of Central South University of Forest & Technology, 35(7), 37–44. 10.14067/j.cnki.1673-923x.2015.07.008 [In Chinese with English Abstract]

[ece35513-bib-0061] Vranckx, G. , Mergeay, J. , Cox, K. , Muys, B. , Jacquemyn, H. , & Honnay, O. (2014). Tree density and population size affect pollen flow and mating patterns in small fragmented forest stands of pedunculate oak (*Quercus robur* L.). Forest Ecology and Management, 328, 254–261. 10.1016/j.foreco.2014.05.044

[ece35513-bib-0062] Wang, H. , Sork, V. L. , Wu, J. , & Ge, J. (2010). Effect of patch size and isolation on mating patterns and seed production in an urban population of Chinese pine (*Pinus tabulaeformi*s Carr.). Forest Ecology and Management, 260, 965–974. 10.1016/j.foreco.2010.06.014

[ece35513-bib-0063] Wang, Z. F. , Ye, W. H. , Fu, S. L. , Ren, H. , & Peng, S. L. (2008). Microsatellites reveal unexpected clonal growth and genetically distinct groups in *Cryptocarya chinensis* in fragmented lower subtropical forest, China. Silvae Genetica, 57, 324–332.

[ece35513-bib-0064] Ye, H. G. , Xu, Z. C. , Wu, M. , & Cao, H. (2013). Geomantic woods in Guangzhou. Wuhan, China: Huazhong University of Science and Technology Press.

[ece35513-bib-0065] Zhao, Z. , Guo, J. , Sha, E. , Lin, K. , Zeng, J. , & Xu, J. (2009). Geographic distribution and phenotypic variation of fruit and seed of *Erythrophleum fordii* in China. Chinese Bulletin of Botany, 44(3), 338–344. 10.3969/j.issn.1674-3466.2009.03.011 [In Chinese]

[ece35513-bib-0066] Zhou, M. (2007). Wood quality of furniture in Ming and Qing Dynasty‐*Erythrophleum fordii* . Collectors, 5, 67–74. [In Chinese].

[ece35513-bib-0067] Zhu, P. , Wang, Z. F. , Ye, W. H. , & Cao, H. L. (2013). Maintenance of genetic diversity in a small, isolated population of ancient tree *Erythrophleum fordii* . Journal of Systematics and Evolution, 51(6), 722–730. 10.1111/jse.12047

[ece35513-bib-0068] Zhu, P. , Wang, Z. F. , Ye, W. H. , Cao, H. L. , & Saravanan, T. (2013). Preliminary studies on pollination and mating system of rare and endangered plant *Erythrophleum fordii* Oliv. Journal of Tropical and Subtropical Botany, 21, 38–44. 10.3969/j.issn.1005-3395.2013.01.005

[ece35513-bib-0069] Zhu, P. , Ye, W. H. , Wang, Z. F. , Cao, H. L. , Zhang, M. , Li, L. , & Xiao, W. (2009). Isolation and characterization of ten polymorphic microsatellite in the endangered tree *Erythrophleum fordii* oliv. Conservation Genetics, 10, 1017–1019. 10.1007/s10592-008-9676-0

