# Supplementary material for: Endangered but genetically stable—Erythrophleum fordii within Feng Shui woodlands in suburbanized villages
Source: Ecol Evol. 2019 Sep 10;9(19):10950–63. doi: 10.1002/ece3.5513 (PMC7277784; doi:10.1002/ece3.5513)

**Figure S1.** Spatial distribution of *Erythrophleum fordii* individuals sampled in different *Feng Shui* woodlands and the DH Mountain nature reserve in China. Red: adults; cyan: juveniles; and yellow: seedlings. Only adults are given proportional circles corresponding to their DBH values.


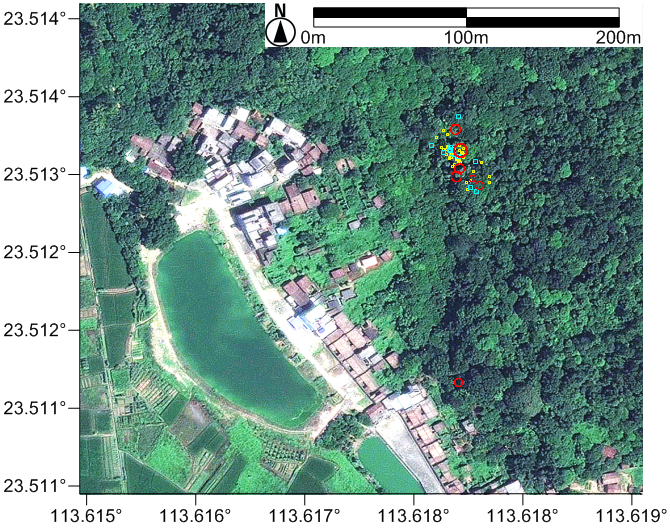


WYG village


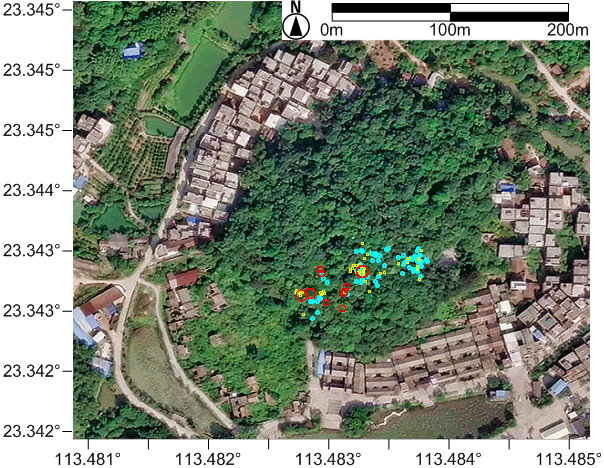


LT village


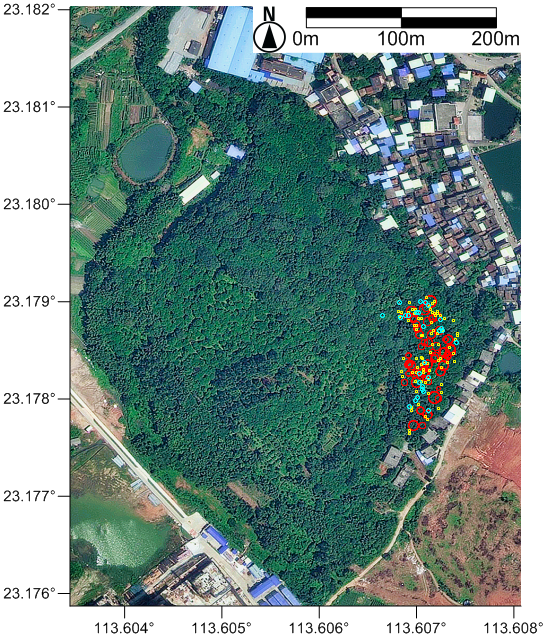


ZPT village


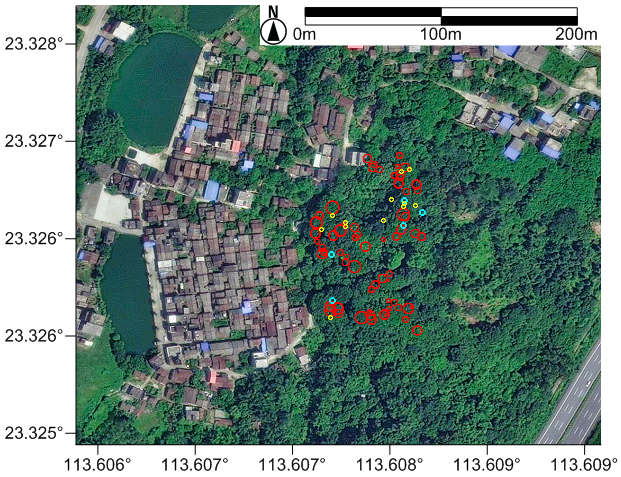


ZL village

SKY village


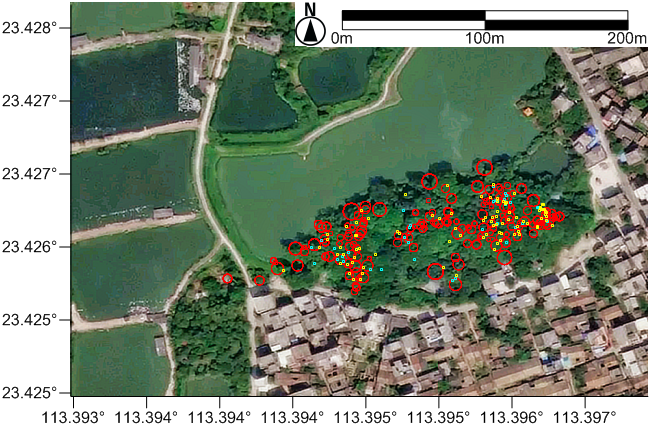


YCG village


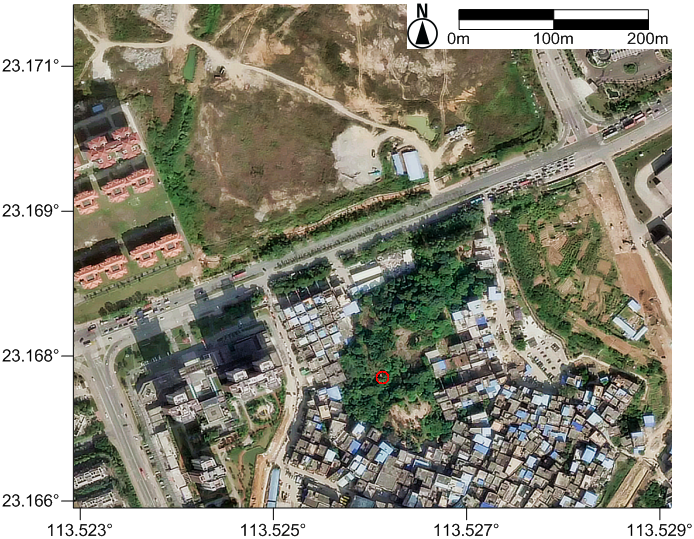


XD village


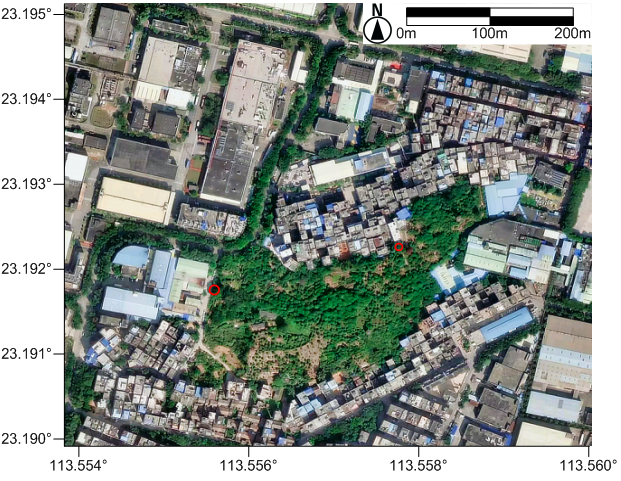


DH Mountain


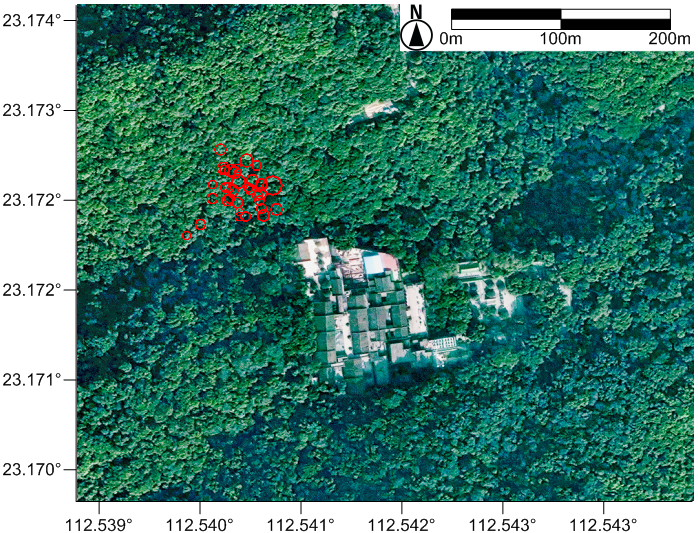

Supplement: Supplementary file 1 [file ECE3-9-10950-s001.docx]
