# Supplementary material for: Endangered but genetically stable—Erythrophleum fordii within Feng Shui woodlands in suburbanized villages
Source: Ecol Evol. 2019 Sep 10;9(19):10950–63. doi: 10.1002/ece3.5513 (PMC7277784; doi:10.1002/ece3.5513)

**Figure S2.** The *Erythrophleum fordii* individual numbered 303 in TB village woodland. The arrows shows some sampled seedlings growing on the roof.


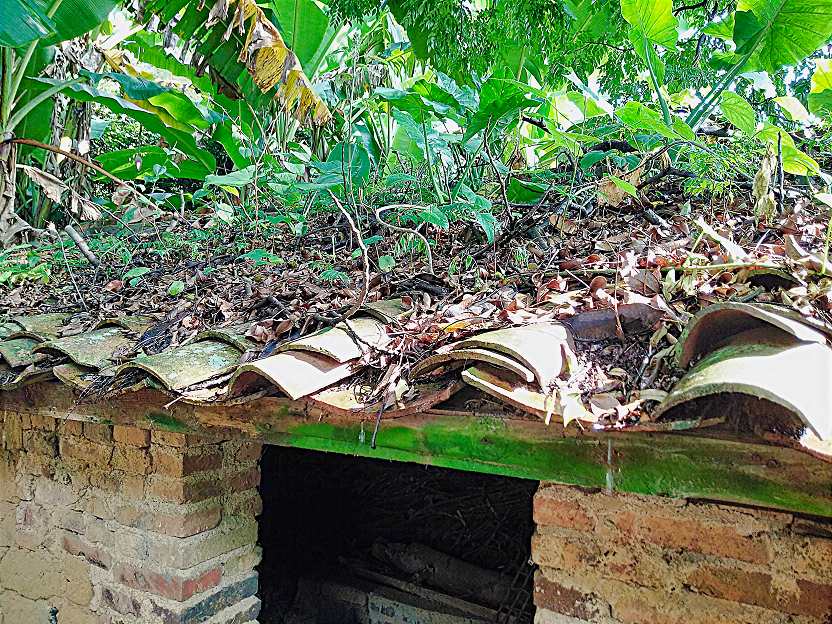

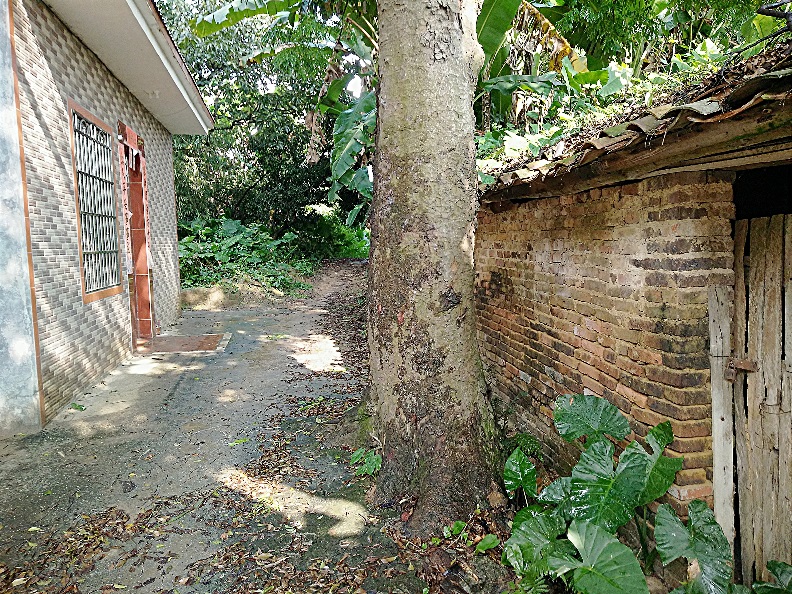

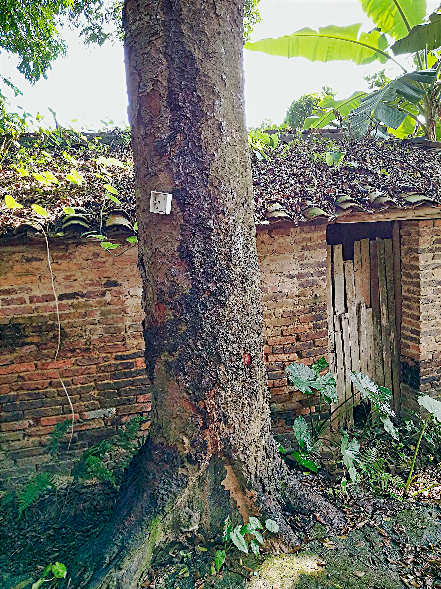

Supplement: Supplementary file 2 [file ECE3-9-10950-s002.docx]
