# Supplementary figures and images for: Endangered but genetically stable—Erythrophleum fordii within Feng Shui woodlands in suburbanized villages
Source: Ecol Evol. 2019 Sep 10;9(19):10950–63. doi: 10.1002/ece3.5513 (PMC7277784; doi:10.1002/ece3.5513)

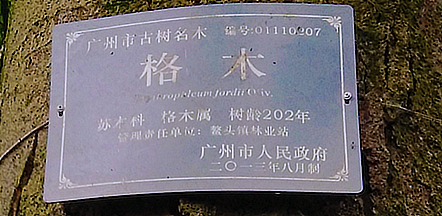

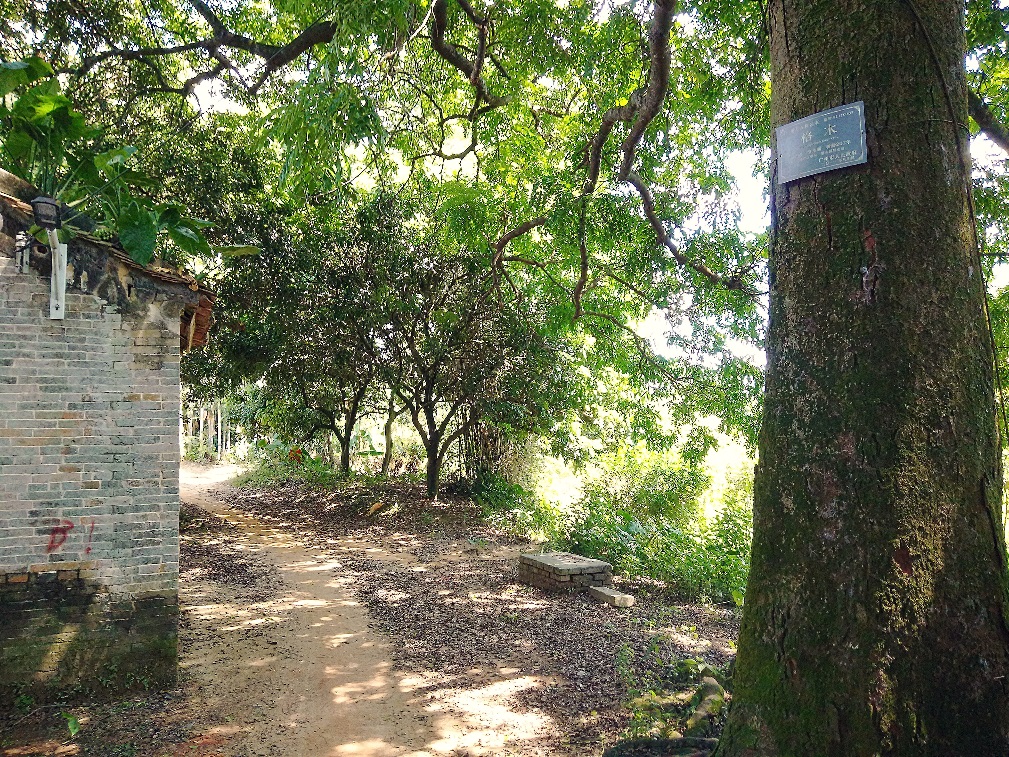
 **Figure S3.** The *Erythrophleum fordii* individual numbered 1 in the TB village woodland.

Supplement: Supplementary file 3 [file ECE3-9-10950-s003.docx]
