# Supplementary material for: Endangered but genetically stable—Erythrophleum fordii within Feng Shui woodlands in suburbanized villages
Source: Ecol Evol. 2019 Sep 10;9(19):10950–63. doi: 10.1002/ece3.5513 (PMC7277784; doi:10.1002/ece3.5513)
Supplement: Supplementary file 4 [file ECE3-9-10950-s004.docx]

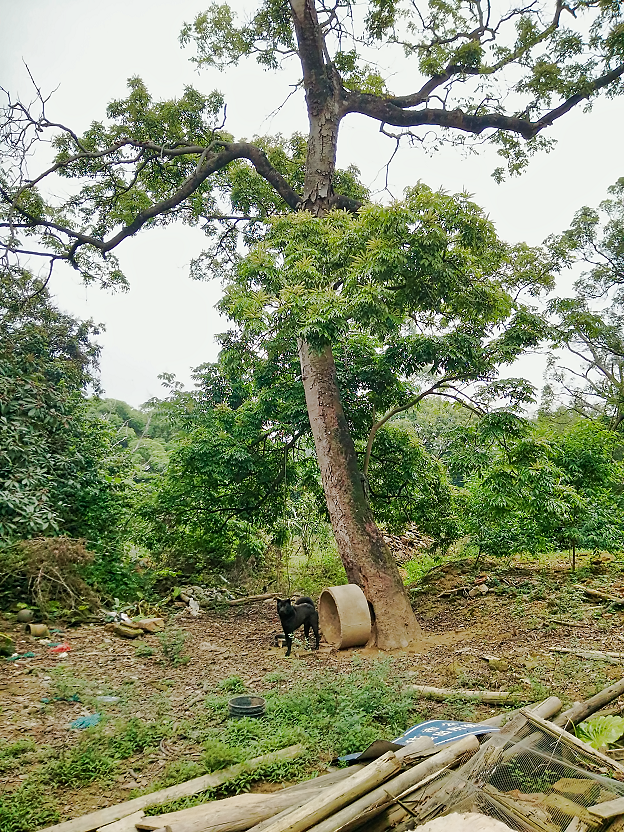

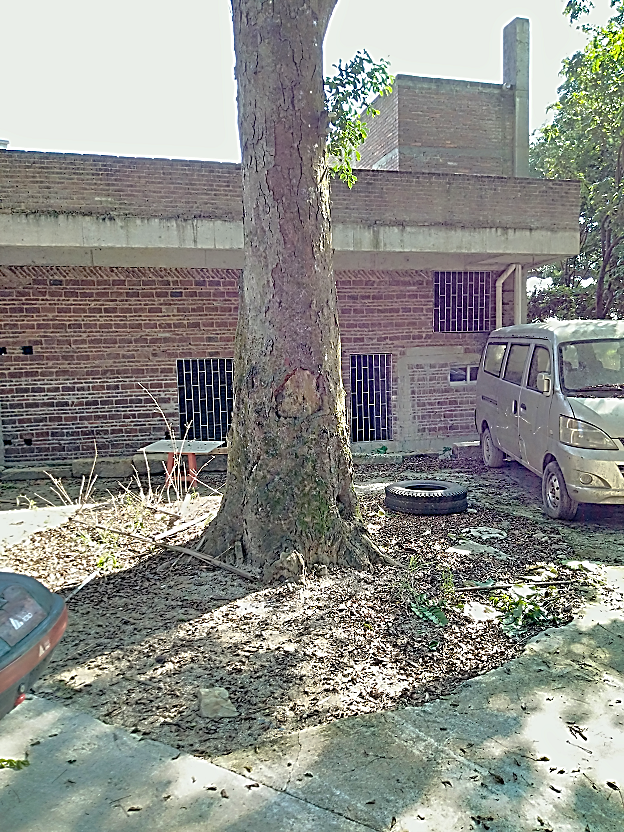
 **Figure S4.** The *Erythrophleum fordii* individuals numbered 35 (left) and 276 (right) in the TB village woodland.
