# Supplementary material for: Endangered but genetically stable—Erythrophleum fordii within Feng Shui woodlands in suburbanized villages
Source: Ecol Evol. 2019 Sep 10;9(19):10950–63. doi: 10.1002/ece3.5513 (PMC7277784; doi:10.1002/ece3.5513)
Supplement: Supplementary file 5 [file ECE3-9-10950-s005.docx]

**Figure S5.** Magnitude of Ln *Pr*(*X*|*K*) and Δ*K* to infer the genetic groups (*K*) in STRUCTURE analysis. Ln *Pr*(*X*|*K*) refers to the mean log likelihood estimate using 20 independent runs for each *K* value. Δ*K* is calculated according to Evanno et al. (2005)
