# Supplementary material for: Endangered but genetically stable—Erythrophleum fordii within Feng Shui woodlands in suburbanized villages
Source: Ecol Evol. 2019 Sep 10;9(19):10950–63. doi: 10.1002/ece3.5513 (PMC7277784; doi:10.1002/ece3.5513)
Supplement: Supplementary file 6 [file ECE3-9-10950-s006.docx]

**Table S1** Pairwise geographic distances between Erythrophleum fordii populations (km)

| Populations | **TB village** | **WYG village** | **LT village** | **ZPT** **village** | **ZL village** | **SKY village** | **YCG village** | **XD village** | **DH Mountain** |
| --- | --- | --- | --- | --- | --- | --- | --- | --- | --- |
| **TB village** |  |  |  |  |  |  |  |  |  |
| **WYG village** | 15.721 |  |  |  |  |  |  |  |  |
| **LT village** | 27.705 | 23.172 |  |  |  |  |  |  |  |
| **ZPT village** | 47.541 | 37.055 | 22.285 |  |  |  |  |  |  |
| **ZL village** | 31.842 | 20.556 | 12.842 | 16.508 |  |  |  |  |  |
| **SKY village** | 20.952 | 24.631 | 12.889 | 35.052 | 24.381 |  |  |  |  |
| **YCG village** | 47.410 | 24.631 | 20.062 | 8.380 | 19.610 | 31.741 |  |  |  |
| **XD village** | 45.037 | 36.115 | 18.400 | 5.491 | 15.947 | 30.786 | 4.051 |  |  |
| **DH Mountain** | 107.687 | 116.250 | 98.175 | 109.028 | 110.383 | 91.692 | 100.742 | 103.756 |  |
