# Supplementary material for: Endangered but genetically stable—Erythrophleum fordii within Feng Shui woodlands in suburbanized villages
Source: Ecol Evol. 2019 Sep 10;9(19):10950–63. doi: 10.1002/ece3.5513 (PMC7277784; doi:10.1002/ece3.5513)
Supplement: Supplementary file 7 [file ECE3-9-10950-s007.docx]

**Table S2**  Characteristics of 16 newly developed microsatellite markers for Erythrophleum fordii*.* The annealing temperature was 53 °C for all loci.

| Locus | Repeat motifs used in RAD sequencing | Primer sequences (5'–3') | Size range (bp) | GENBANK accession no. |
| --- | --- | --- | --- | --- |
| *EF-1* | (GT)_10_ | F: FAM-GGTGGAGGTACGTTGAATTGC  R: ATGTCATAAATGGGTCCGGTG | 150−182 | MH051736 |
| *EF-4* | (AT)_9_ | F: FAM-GCTGATAAGGAGATAGGCCAAATAC  R: TCTCTTCCTAAGTCTCAACATGC | 255−263 | MH051737 |
| *EF-5* | (TC)_5_TA(TC)_9_ | F: FAM-CCATTGGAGGGTTTGAGCG  R: AGCTTCAGTTATCCTCCAGCG | 267−285 | MH051738 |
| *EF-6* | (AC)_14_ | F: FAM-GCCATCTGGCATAGGTGTC  R: TGAAGTTGATGGATTTCCACAGG | 161−167 | MH051739 |
| *EF-7* | (AAT)_9_ | F: FAM-GCCAAAGCAAATAACAACGC  R: CTTGCGCTGGGAGTAAAGC | 235−241 | MH051740 |
| *EF-9* | (CTT)_7_ | F: FAM-GCTTCTTTGTCCGCCCTTG  R: ACAGCGAAGAAAGCATCCAC | 264−276 | MH051741 |
| *EF-10* | (TA)_10_ | F: FAM-CAAGTGAAGAATGAATGCCCTC  R: AATTCTCTTATCTCGATTGGTTAGG | 276−298 | MH051742 |
| *EF-19* | (AT)_11_ | F: FAM-ACGGTGTGTTCGATAGAATGG  R: TGTGGACATTATGCAGTTGGTG | 166−170 | MH051743 |
| *EF-20* | (AT)_8_ | F: FAM-CAGAATCAGAACAGCATGTTTCC  R: GGAACAAGAGTTCCTGCGG | 226−228 | MH051744 |
| *EF-26* | (AG)_13_ | F: FAM-CGAGGAAAGACCGGAGGC  R: CATCTTACCTTGGCCACGTC | 150−166 | MH051745 |
| *EF-28* | (TA)_8_ | F: FAM-CCTTGACTGCCATTAGACCC  R: TGGTGCTTGTATGAAGAGGAC | 226−244 | MH051746 |
| *EF-29* | (AT)_13_ | F: FAM-AGTGTCTAAACTGATCAAACCAAGG  R: TGCAGGAGGGTTGCAATATC | 161−181 | MH051747 |
| *EF-30* | (AT)_11_ | F: FAM-ACAGTTCAGCACTTATGTTATACCG  R: ATTCAGAAGTTTGGGAACACG | 191−209 | MH051748 |
| *EF-32* | (AT)_11_ | F: FAM-AAGGCACGAGAGCTATGAC  R: TCTAGAGGATTCTTTGCCTTTGG | 150−156 | MH051749 |
| *EF-33* | (AT)_12_ | F: FAM-AACGCTTCAATTGGTGACTG  R: TTGCAGACGTTTCCGCTTG | 365−427 | MH051750 |
| *EF-35* | (AT)_16_ | F: FAM-AGCTTCATATGTTTCTGCGTGT  R: CGCCAGCAATGCAGTTAAT | 236−256 | MH051751 |
