# Supplementary material for: Endangered but genetically stable—Erythrophleum fordii within Feng Shui woodlands in suburbanized villages
Source: Ecol Evol. 2019 Sep 10;9(19):10950–63. doi: 10.1002/ece3.5513 (PMC7277784; doi:10.1002/ece3.5513)
Supplement: Supplementary file 8 [file ECE3-9-10950-s008.doc]

Table S3 Allelic variation in Erythrophleum fordii populations

| **Tangbei** | | | | | | | | | | | | | | | | | | | |
| --- | --- | --- | --- | --- | --- | --- | --- | --- | --- | --- | --- | --- | --- | --- | --- | --- | --- | --- | --- |
| Locus | All individuals  (346 individuals) | | | |  | Adult  (18 individuals) | | | |  | Juvenile  (71 individuals) | | | |  | Seedling  (257 individuals) | | | |
|  | *A* | *H_O_* | *H_E_* | *f* |  | *A* | *H_O_* | *H_E_* | *f* |  | *A* | *H_O_* | *H_E_* | *f* |  | *A* | *H_O_* | *H_E_* | *f* |
| *EF-1* | 3 | 0.4017 | 0.4041 | 0.0059 |  | 3 | 0.1667 | 0.2524 | 0.3462 |  | 3 | 0.4225 | 0.4404 | 0.0409 |  | 3 | 0.4125 | 0.4021 | -0.0259 |
| *EF-4* | 2 | 0.3382 | 0.3180 | -0.0634 |  | 2 | 0.3889 | 0.3222 | -0.2143 |  | 2 | 0.3521 | 0.3437 | -0.0246 |  | 2 | 0.3307 | 0.3116 | -0.0615 |
| *EF-5* | 3 | 0.2312 | 0.2143 | -0.0791 |  | 2 | 0.1667 | 0.1571 | -0.0625 |  | 3 | 0.2394 | 0.2153 | -0.1132 |  | 2 | 0.2335 | 0.2184 | -0.0689 |
| *EF-6* | 2 | 0.4566 | 0.4140 | -0.1032 |  | 2 | 0.2778 | 0.3857 | 0.2857 |  | 2 | 0.4225 | 0.3948 | -0.0709 |  | 2 | 0.4786 | 0.4221 | -0.1343 |
| *EF-7* | 2 | 0.5116 | 0.4510 | -0.1344 |  | 2 | 0.6667 | 0.5079 | -0.3247 |  | 2 | 0.4930 | 0.4712 | -0.0466 |  | 2 | 0.5058 | 0.4409 | -0.1476 |
| *EF-9* | 2 | 0.4971 | 0.5006 | 0.0069 |  | 2 | 0.7222 | 0.5127 | -0.4258 |  | 2 | 0.4225 | 0.4507 | 0.0629 |  | 2 | 0.5019 | 0.4941 | -0.0158 |
| *EF-10* | 5 | 0.7977 | 0.7244 | -0.1013 |  | 4 | 0.7222 | 0.7476 | 0.0349 |  | 4 | 0.8028 | 0.7127 | -0.1274 |  | 5 | 0.8016 | 0.7267 | -0.1033 |
| *EF-19* | 3 | 0.5434 | 0.5053 | -0.0753 |  | 3 | 0.5556 | 0.5603 | 0.0087 |  | 3 | 0.5070 | 0.4139 | -0.2269 |  | 3 | 0.5525 | 0.5228 | -0.0570 |
| *EF-20* | 2 | 0.0145 | 0.0144 | -0.0058 |  | 2 | 0.0556 | 0.0556 | − |  | 2 | 0.0141 | 0.0141 | − |  | 2 | 0.0117 | 0.0116 | -0.0039 |
| *EF-26* | 2 | 0.1098 | 0.1293 | 0.1507 |  | 2 | 0.3333 | 0.4127 | 0.1969 |  | 2 | 0.1408 | 0.1558 | 0.0968 |  | 2 | 0.0856 | 0.0962 | 0.1107 |
| *EF-28* | 5 | 0.6272 | 0.7064 | 0.1123* |  | 4 | 0.7222 | 0.7000 | -0.0327 |  | 5 | 0.5634 | 0.6773 | 0.1691 |  | 5 | 0.6381 | 0.6972 | 0.0849 |
| *EF-29* | 6 | 0.4783 | 0.5294 | 0.0967 |  | 4 | 0.5000 | 0.5698 | 0.1257 |  | 4 | 0.6571 | 0.6223 | -0.0564 |  | 6 | 0.4280 | 0.4921 | 0.1304 |
| *EF-30* | 5 | 0.6243 | 0.5953 | -0.0488 |  | 4 | 0.7222 | 0.6381 | -0.1362 |  | 5 | 0.6761 | 0.6598 | -0.0249 |  | 5 | 0.6031 | 0.5732 | -0.0523 |
| *EF-32* | 3 | 0.4810 | 0.6092 | 0.2105* |  | 3 | 0.6111 | 0.6857 | 0.1116 |  | 3 | 0.5775 | 0.6705 | 0.1396 |  | 3 | 0.4449 | 0.5654 | 0.2134* |
| *EF-33* | 11 | 0.6214 | 0.6049 | -0.0273 |  | 6 | 0.6111 | 0.5746 | -0.0655 |  | 11 | 0.7606 | 0.7215 | -0.0545 |  | 7 | 0.5837 | 0.5662 | -0.0308 |
| *EF-35* | 5 | 0.4075 | 0.4214 | 0.0329 |  | 4 | 0.4444 | 0.3825 | -0.1674 |  | 5 | 0.2535 | 0.2552 | 0.0067 |  | 5 | 0.4475 | 0.4640 | 0.0356 |
| *Gm1048* | 7 | 0.7370 | 0.7146 | -0.0314 |  | 5 | 0.7222 | 0.7492 | 0.0370 |  | 7 | 0.7606 | 0.7633 | 0.0036 |  | 7 | 0.7315 | 0.6889 | -0.062 |
| *Gm2062* | 2 | 0.5116 | 0.4967 | -0.03 |  | 2 | 0.5556 | 0.4127 | -0.3600 |  | 2 | 0.4930 | 0.4936 | 0.0012 |  | 2 | 0.5136 | 0.4997 | -0.0279 |
| *Gm2024* | 9 | 0.6590 | 0.6623 | 0.0051 |  | 6 | 0.5556 | 0.7587 | 0.2735 |  | 7 | 0.8732 | 0.7290 | -0.1996 |  | 7 | 0.6070 | 0.6243 | 0.0277 |
| *Gm2065* | 9 | 0.8092 | 0.7907 | -0.0234 |  | 7 | 0.7778 | 0.7873 | 0.0124 |  | 9 | 0.7183 | 0.7791 | 0.0786 |  | 9 | 0.8366 | 0.7932 | -0.0548 |
| *Gm4058* | 5 | 0.7478 | 0.6894 | -0.0849 |  | 5 | 0.6667 | 0.7127 | 0.0664 |  | 5 | 0.7887 | 0.6788 | -0.1634 |  | 5 | 0.7422 | 0.6877 | -0.0794 |
|  | **Wayaogang** | | | | | | | | | | | | | | | | | | |
| Locus | All individuals  (60 individuals) | | | |  | Adult+Juvenile  (18 individuals) | | | |  | Seedling  (42 individuals) | | | |  |  | | | |
|  | *A* | *H_O_* | *H_E_* | *f* |  | *A* | *H_O_* | *H_E_* | *f* |  | *A* | *H_O_* | *H_E_* | *f* |  |  |  |  |  |
| *EF-1* | 2 | 0.3500 | 0.3125 | -0.1213 |  | 2 | 0.1667 | 0.2460 | 0.3289 |  | 2 | 0.4286 | 0.3408 | -0.2615 |  |  |  |  |  |
| *EF-4* | 2 | 0.0833 | 0.0805 | -0.0351 |  | 2 | 0.0556 | 0.0556 | − |  | 2 | 0.0952 | 0.0918 | -0.0380 |  |  |  |  |  |
| *EF-5* | 2 | 0.0500 | 0.0492 | -0.0172 |  | 1 | 0.0000 | 0.0000 | − |  | 2 | 0.0714 | 0.0697 | -0.0250 |  |  |  |  |  |
| *EF-6* | 2 | 0.4833 | 0.4301 | -0.1249 |  | 2 | 0.4444 | 0.4571 | 0.0286 |  | 2 | 0.5000 | 0.4231 | -0.1843 |  |  |  |  |  |
| *EF-7* | 2 | 0.5333 | 0.4768 | -0.1198 |  | 2 | 0.6667 | 0.5079 | -0.3247 |  | 2 | 0.4762 | 0.4647 | -0.0250 |  |  |  |  |  |
| *EF-9* | 2 | 0.2333 | 0.2078 | -0.1238 |  | 2 | 0.1667 | 0.1571 | -0.0625 |  | 2 | 0.2619 | 0.2303 | -0.1389 |  |  |  |  |  |
| *EF-10* | 4 | 0.8000 | 0.6620 | -0.2105 |  | 4 | 0.7778 | 0.6683 | -0.1695 |  | 4 | 0.8095 | 0.6552 | -0.2391 |  |  |  |  |  |
| *EF-19* | 3 | 0.4500 | 0.3618 | -0.2465 |  | 2 | 0.2778 | 0.2460 | -0.1333 |  | 3 | 0.5238 | 0.4076 | -0.2895 |  |  |  |  |  |
| *EF-20* | 2 | 0.0333 | 0.0331 | -0.0085 |  | 1 | 0.0000 | 0.0000 | − |  | 2 | 0.0476 | 0.0470 | -0.0123 |  |  |  |  |  |
| *EF-26* | 2 | 0.1667 | 0.1541 | -0.0826 |  | 2 | 0.1667 | 0.1571 | -0.0625 |  | 2 | 0.1667 | 0.1546 | -0.0789 |  |  |  |  |  |
| *EF-28* | 5 | 0.1356 | 0.6868 | 0.8039* |  | 4 | 0.2222 | 0.6571 | 0.6683* |  | 5 | 0.0976 | 0.7037 | 0.8628* |  |  |  |  |  |
| *EF-29* | 4 | 0.5833 | 0.4794 | -0.2190 |  | 2 | 0.7222 | 0.4746 | -0.5455 |  | 4 | 0.5238 | 0.4839 | -0.0835 |  |  |  |  |  |
| *EF-30* | 4 | 0.6000 | 0.5032 | -0.1943 |  | 2 | 0.5556 | 0.5143 | -0.0828 |  | 4 | 0.6190 | 0.4885 | -0.2713 |  |  |  |  |  |
| *EF-33* | 5 | 0.8167 | 0.6711 | -0.2191 |  | 5 | 0.7778 | 0.6746 | -0.1582 |  | 4 | 0.8333 | 0.6756 | -0.2371 |  |  |  |  |  |
| *EF-35* | 4 | 0.5167 | 0.4783 | -0.0810 |  | 3 | 0.6111 | 0.5143 | -0.1949 |  | 4 | 0.4762 | 0.4667 | -0.0205 |  |  |  |  |  |
| *Gm1048* | 5 | 0.8475 | 0.6257 | -0.3586* |  | 4 | 0.7222 | 0.6079 | -0.1946 |  | 5 | 0.9024 | 0.6399 | -0.4176* |  |  |  |  |  |
| *Gm2062* | 2 | 0.5833 | 0.5041 | -0.1588 |  | 2 | 0.5556 | 0.5079 | -0.0968 |  | 2 | 0.5952 | 0.5057 | -0.1795 |  |  |  |  |  |
| *Gm2024* | 5 | 0.4667 | 0.4758 | 0.0193 |  | 4 | 0.6111 | 0.5603 | -0.0936 |  | 5 | 0.4048 | 0.4375 | 0.0756 |  |  |  |  |  |
| *Gm2065* | 8 | 0.7667 | 0.6590 | -0.1651 |  | 7 | 0.7778 | 0.6651 | -0.1753 |  | 8 | 0.7619 | 0.6598 | -0.1570 |  |  |  |  |  |
| *Gm4058* | 4 | 0.4915 | 0.4484 | -0.0972 |  | 4 | 0.6111 | 0.5984 | -0.0219 |  | 4 | 0.4390 | 0.3719 | -0.1832 |  |  |  |  |  |
|  |  | | | | | | | | | | | | | | | | | | |
|  |  | | | | | | | | | | | | | | | | | | |
|  | **Liantang** | | | | | | | | | | | | | | | | | | |
| Locus | All individuals  (130 individuals) | | | |  | Juvenile  (75 individuals) | | | |  | Seedling  ( 46 individuals) | | | |  |  | | | |
|  | *A* | *H_O_* | *H_E_* | *f* |  | *A* | *H_O_* | *H_E_* | *f* |  | *A* | *H_O_* | *H_E_* | *f* |  |  |  |  |  |
| *EF-1* | 2 | 0.2308 | 0.2049 | -0.1266 |  | 2 | 0.2400 | 0.2126 | -0.1298 |  | 2 | 0.2391 | 0.2129 | -0.1250 |  |  |  |  |  |
| *EF-4* | 3 | 0.3769 | 0.3582 | -0.0526 |  | 3 | 0.3467 | 0.3632 | 0.0459 |  | 2 | 0.4348 | 0.3679 | -0.1842 |  |  |  |  |  |
| *EF-5* | 3 | 0.4923 | 0.4683 | -0.0514 |  | 2 | 0.4133 | 0.4429 | 0.0671 |  | 3 | 0.5652 | 0.5029 | -0.1255 |  |  |  |  |  |
| *EF-6* | 3 | 0.5846 | 0.6388 | 0.0851 |  | 3 | 0.6000 | 0.6066 | 0.0110 |  | 3 | 0.5870 | 0.6424 | 0.0872 |  |  |  |  |  |
| *EF-7* | 2 | 0.1769 | 0.1868 | 0.0533 |  | 2 | 0.1733 | 0.1812 | 0.0437 |  | 2 | 0.1739 | 0.1605 | -0.0843 |  |  |  |  |  |
| *EF-9* | 2 | 0.4462 | 0.4715 | 0.0540 |  | 2 | 0.4133 | 0.4600 | 0.1022 |  | 2 | 0.4565 | 0.4861 | 0.0616 |  |  |  |  |  |
| *EF-10* | 5 | 0.6538 | 0.6218 | -0.0517 |  | 4 | 0.6933 | 0.6252 | -0.1097 |  | 4 | 0.5870 | 0.5936 | 0.0114 |  |  |  |  |  |
| *EF-19* | 3 | 0.5846 | 0.5946 | 0.0168 |  | 3 | 0.6133 | 0.5524 | -0.1111 |  | 3 | 0.5217 | 0.6316 | 0.1756 |  |  |  |  |  |
| *EF-20* | 2 | 0.0462 | 0.0453 | -0.0198 |  | 2 | 0.0400 | 0.0395 | -0.0137 |  | 2 | 0.0435 | 0.0430 | -0.0112 |  |  |  |  |  |
| *EF-26* | 2 | 0.0538 | 0.0526 | -0.0238 |  | 2 | 0.0800 | 0.0773 | -0.0350 |  | 2 | 0.0217 | 0.0217 | − |  |  |  |  |  |
| *EF-28* | 5 | 0.4806 | 0.7327 | 0.3449* |  | 5 | 0.4054 | 0.7300 | 0.4463* |  | 5 | 0.5652 | 0.7043 | 0.1992 |  |  |  |  |  |
| *EF-29* | 6 | 0.6923 | 0.7708 | 0.1022 |  | 6 | 0.7467 | 0.7776 | 0.0401 |  | 5 | 0.5870 | 0.7499 | 0.2192 |  |  |  |  |  |
| *EF-30* | 5 | 0.4308 | 0.4111 | -0.0480 |  | 5 | 0.5200 | 0.4688 | -0.1100 |  | 4 | 0.3261 | 0.3108 | -0.0498 |  |  |  |  |  |
| *EF-33* | 9 | 0.7231 | 0.6912 | -0.0463 |  | 7 | 0.7067 | 0.6500 | -0.0878 |  | 7 | 0.7609 | 0.7463 | -0.0197 |  |  |  |  |  |
| *EF-35* | 5 | 0.4308 | 0.4457 | 0.0336 |  | 5 | 0.5333 | 0.5323 | -0.0019 |  | 4 | 0.2609 | 0.2850 | 0.0855 |  |  |  |  |  |
| *Gm1048* | 5 | 0.7385 | 0.6982 | -0.0579 |  | 5 | 0.7467 | 0.6750 | -0.1070 |  | 5 | 0.6957 | 0.7097 | 0.0201 |  |  |  |  |  |
| *Gm2062* | 2 | 0.4769 | 0.4495 | -0.0612 |  | 2 | 0.4800 | 0.4381 | -0.0963 |  | 2 | 0.5000 | 0.4766 | -0.0497 |  |  |  |  |  |
| *Gm2024* | 6 | 0.7385 | 0.7288 | -0.0133 |  | 5 | 0.7200 | 0.7303 | 0.0142 |  | 5 | 0.8261 | 0.7143 | -0.1585 |  |  |  |  |  |
| *Gm2065* | 8 | 0.8308 | 0.7938 | -0.0467 |  | 8 | 0.8400 | 0.7882 | -0.0662 |  | 6 | 0.8043 | 0.7855 | -0.0243 |  |  |  |  |  |
| *Gm4058* | 5 | 0.7462 | 0.6971 | -0.0707 |  | 5 | 0.7067 | 0.7076 | 0.0013 |  | 5 | 0.7609 | 0.6820 | -0.1170 |  |  |  |  |  |
|  |  | | | | | | | | | | | | | | | | | | |
|  |  | | | | | | | | | | | | | | | | | | |
|  | **Zhongbotou** | | | | | | | | | | | | | | | | | | |
| Locus | All individuals  (164 individuals) | | | |  | Adult  (59 individuals) | | | |  | Juvenile  (27 individuals) | | | |  | Seedling  (78 individuals) | | | |
|  | *A* | *H_O_* | *H_E_* | *f* |  | *A* | *H_O_* | *H_E_* | *f* |  | *A* | *H_O_* | *H_E_* | *f* |  | *A* | *H_O_* | *H_E_* | *f* |
| *EF-1* | 2 | 0.2927 | 0.2998 | 0.0240 |  | 2 | 0.2542 | 0.2238 | -0.1373 |  | 2 | 0.2222 | 0.3913 | 0.4368 |  | 2 | 0.3462 | 0.3205 | -0.0806 |
| *EF-4* | 2 | 0.4695 | 0.4178 | -0.1241 |  | 2 | 0.4068 | 0.3987 | -0.0205 |  | 2 | 0.5556 | 0.4088 | -0.3684 |  | 2 | 0.4872 | 0.4384 | -0.1121 |
| *EF-5* | 3 | 0.4146 | 0.3777 | -0.0982 |  | 2 | 0.3559 | 0.3559 | 0.0000 |  | 2 | 0.4444 | 0.3522 | -0.2683 |  | 3 | 0.4487 | 0.4054 | -0.1077 |
| *EF-6* | 2 | 0.3659 | 0.3297 | -0.1101 |  | 2 | 0.3051 | 0.2839 | -0.0752 |  | 2 | 0.2963 | 0.2572 | -0.1556 |  | 2 | 0.4359 | 0.3838 | -0.1368 |
| *EF-7* | 2 | 0.5366 | 0.4979 | -0.0780 |  | 2 | 0.5593 | 0.4991 | -0.1219 |  | 2 | 0.3704 | 0.5087 | 0.2758 |  | 2 | 0.5769 | 0.4965 | -0.1631 |
| *EF-9* | 2 | 0.3659 | 0.3636 | -0.0062 |  | 2 | 0.3390 | 0.3268 | -0.0376 |  | 2 | 0.4815 | 0.4088 | -0.1818 |  | 2 | 0.3462 | 0.3774 | 0.0833 |
| *EF-10* | 5 | 0.7195 | 0.6654 | -0.0816 |  | 5 | 0.8305 | 0.6726 | -0.2373 |  | 4 | 0.5926 | 0.6352 | 0.0683 |  | 4 | 0.6795 | 0.6747 | -0.0072 |
| *EF-19* | 3 | 0.6402 | 0.5708 | -0.1220 |  | 3 | 0.6441 | 0.5447 | -0.1843 |  | 3 | 0.6296 | 0.6226 | -0.0114 |  | 3 | 0.6410 | 0.5751 | -0.1155 |
| *EF-20* | 2 | 0.0854 | 0.0820 | -0.0415 |  | 2 | 0.0508 | 0.0500 | -0.0175 |  | 2 | 0.1852 | 0.1712 | -0.0833 |  | 2 | 0.0769 | 0.0744 | -0.0336 |
| *EF-26* | 2 | 0.0793 | 0.0764 | -0.0382 |  | 2 | 0.0508 | 0.0500 | -0.0175 |  | 2 | 0.0370 | 0.0370 | − |  | 2 | 0.1154 | 0.1094 | -0.0548 |
| *EF-28* | 6 | 0.4085 | 0.6297 | 0.3519 |  | 6 | 0.4068 | 0.5812 | 0.3019 |  | 6 | 0.4074 | 0.7058 | 0.4274 |  | 6 | 0.4103 | 0.6298 | 0.3500 |
| *EF-29* | 6 | 0.7439 | 0.7287 | -0.0209 |  | 6 | 0.6949 | 0.6978 | 0.0042 |  | 6 | 0.8148 | 0.7890 | -0.0334 |  | 6 | 0.7564 | 0.7323 | -0.0332 |
| *EF-30* | 5 | 0.5183 | 0.5105 | -0.0153 |  | 5 | 0.4068 | 0.4223 | 0.0370 |  | 5 | 0.4815 | 0.5395 | 0.1094 |  | 5 | 0.6154 | 0.5630 | -0.0937 |
| *EF-33* | 8 | 0.7744 | 0.7404 | -0.0461 |  | 8 | 0.8136 | 0.7164 | -0.1370 |  | 6 | 0.7407 | 0.7456 | 0.0067 |  | 8 | 0.7564 | 0.7521 | -0.0058 |
| *EF-35* | 4 | 0.5732 | 0.5005 | -0.1457 |  | 3 | 0.4746 | 0.4272 | -0.1119 |  | 4 | 0.5556 | 0.5639 | 0.0152 |  | 4 | 0.6538 | 0.5308 | -0.2337 |
| *Gm1048* | 5 | 0.8171 | 0.7726 | -0.0577 |  | 5 | 0.8644 | 0.7598 | -0.1390 |  | 5 | 0.7407 | 0.7575 | 0.0226 |  | 5 | 0.8077 | 0.7719 | -0.0467 |
| *Gm2062* | 2 | 0.4146 | 0.4431 | 0.0643 |  | 2 | 0.4915 | 0.4759 | -0.0332 |  | 2 | 0.3333 | 0.3305 | -0.0086 |  | 2 | 0.3846 | 0.4473 | 0.1409 |
| *Gm2024* | 6 | 0.3049 | 0.3268 | 0.0672 |  | 4 | 0.2034 | 0.2450 | 0.1709 |  | 5 | 0.4444 | 0.3843 | -0.1599 |  | 4 | 0.3333 | 0.3675 | 0.0935 |
| *Gm2065* | 8 | 0.8232 | 0.7743 | -0.0633 |  | 7 | 0.8814 | 0.7955 | -0.1090 |  | 5 | 0.6296 | 0.6737 | 0.0665 |  | 7 | 0.8462 | 0.7675 | -0.1032 |
| *Gm4058* | 5 | 0.6341 | 0.6325 | -0.0026 |  | 5 | 0.6610 | 0.6432 | -0.0279 |  | 5 | 0.7407 | 0.6415 | -0.1581 |  | 5 | 0.5769 | 0.6177 | 0.0664 |
|  |  | | | | | | | | | | | | | | | | | | |
|  |  | | | | | | | | | | | | | | | | | | |
|  | **Zhongling** | | | | | | | | | | | | | | | | | | |
| Locus | All individuals  (82 individuals) | | | |  | Adult  (65 individuals) | | | |  | Juvenile+Seedling  (17 individuals) | | | |  |  | | | |
|  | *A* | *H_O_* | *H_E_* | *f* |  | *A* | *H_O_* | *H_E_* | *f* |  | *A* | *H_O_* | *H_E_* | *f* |  |  |  |  |  |
| *EF-1* | 2 | 0.0366 | 0.0361 | -0.0125 |  | 2 | 0.0462 | 0.0454 | -0.0159 |  | 1 | 0.0000 | 0.0000 | − |  |  |  |  |  |
| *EF-4* | 2 | 0.5000 | 0.5030 | 0.0060 |  | 2 | 0.4615 | 0.5038 | 0.0844 |  | 2 | 0.6471 | 0.5152 | -0.2662 |  |  |  |  |  |
| *EF-5* | 3 | 0.0610 | 0.0598 | -0.0202 |  | 3 | 0.0769 | 0.0750 | -0.0256 |  | 1 | 0.0000 | 0.0000 | − |  |  |  |  |  |
| *EF-6* | 3 | 0.4390 | 0.4045 | -0.0858 |  | 3 | 0.3846 | 0.3694 | -0.0417 |  | 3 | 0.6471 | 0.5330 | -0.2222 |  |  |  |  |  |
| *EF-7* | 2 | 0.3780 | 0.4114 | 0.0816 |  | 2 | 0.3538 | 0.3965 | 0.1084 |  | 2 | 0.4706 | 0.4706 | 0.0000 |  |  |  |  |  |
| *EF-9* | 2 | 0.5244 | 0.4970 | -0.0555 |  | 2 | 0.5385 | 0.4867 | -0.1073 |  | 2 | 0.4706 | 0.4991 | 0.0588 |  |  |  |  |  |
| *EF-10* | 4 | 0.7805 | 0.6742 | -0.1588 |  | 4 | 0.8000 | 0.6779 | -0.1818 |  | 4 | 0.7059 | 0.6720 | -0.0521 |  |  |  |  |  |
| *EF-19* | 3 | 0.6098 | 0.5431 | -0.1236 |  | 3 | 0.6154 | 0.5324 | -0.1573 |  | 3 | 0.5882 | 0.5579 | -0.0561 |  |  |  |  |  |
| *EF-20* | 2 | 0.2683 | 0.2849 | 0.0586 |  | 2 | 0.3077 | 0.3034 | -0.0143 |  | 2 | 0.1176 | 0.2139 | 0.4576 |  |  |  |  |  |
| *EF-26* | 2 | 0.3049 | 0.2929 | -0.0411 |  | 2 | 0.3231 | 0.2935 | -0.1016 |  | 2 | 0.2353 | 0.2995 | 0.2195 |  |  |  |  |  |
| *EF-28* | 5 | 0.3902 | 0.7029 | 0.4463* |  | 5 | 0.4308 | 0.7233 | 0.4063* |  | 5 | 0.2353 | 0.5544 | 0.5831 |  |  |  |  |  |
| *EF-29* | 6 | 0.7317 | 0.6407 | -0.1430 |  | 6 | 0.7385 | 0.6433 | -0.1493 |  | 5 | 0.7059 | 0.6417 | -0.1034 |  |  |  |  |  |
| *EF-30* | 5 | 0.7683 | 0.6891 | -0.1157 |  | 5 | 0.7692 | 0.6661 | -0.1563 |  | 5 | 0.7647 | 0.7522 | -0.0171 |  |  |  |  |  |
| *EF-33* | 8 | 0.9024 | 0.7529 | -0.2001 |  | 8 | 0.9077 | 0.7349 | -0.2374 |  | 6 | 0.8824 | 0.8093 | -0.0934* |  |  |  |  |  |
| *EF-35* | 5 | 0.6098 | 0.6098 | 0.0000 |  | 5 | 0.5692 | 0.5595 | -0.0176 |  | 4 | 0.7647 | 0.6845 | -0.1213 |  |  |  |  |  |
| *Gm1048* | 4 | 0.8659 | 0.7203 | -0.2035 |  | 4 | 0.8462 | 0.7119 | -0.1904 |  | 4 | 0.9412 | 0.7469 | -0.2705 |  |  |  |  |  |
| *Gm2062* | 2 | 0.4268 | 0.3647 | -0.1715 |  | 2 | 0.4462 | 0.3660 | -0.2211 |  | 2 | 0.3529 | 0.3708 | 0.0495 |  |  |  |  |  |
| *Gm2024* | 5 | 0.5488 | 0.5208 | -0.0541 |  | 4 | 0.5385 | 0.5265 | -0.0228 |  | 5 | 0.5882 | 0.4991 | -0.1852 |  |  |  |  |  |
| *Gm2065* | 5 | 0.7805 | 0.7031 | -0.1108 |  | 4 | 0.7846 | 0.7004 | -0.1213 |  | 5 | 0.7647 | 0.6364 | -0.2093 |  |  |  |  |  |
| *Gm4058* | 4 | 0.7927 | 0.7233 | -0.0965 |  | 4 | 0.8000 | 0.7246 | -0.1049 |  | 4 | 0.7647 | 0.7130 | -0.0749 |  |  |  |  |  |
|  |  | | | | | | | | | | | | | | | | | | |
|  |  | | | | | | | | | | | | | | | | | | |
|  | **Shuikouying** | | | | | | | | | | | | | | | | | | |
| Locus | All individuals  (276 individuals) | | | |  | Adult  (155 individuals) | | | |  | Juvenile  (34 individuals) | | | |  | Seedling  (87 individuals) | | | |
|  | *A* | *H_O_* | *H_E_* | *f* |  | *A* | *H_O_* | *H_E_* | *f* |  | *A* | *H_O_* | *H_E_* | *f* |  | *A* | *H_O_* | *H_E_* | *f* |
| *EF-1* | 2 | 0.0399 | 0.0391 | -0.0185 |  | 2 | 0.0323 | 0.0318 | -0.0132 |  | 2 | 0.0588 | 0.0579 | -0.0154 |  | 2 | 0.0460 | 0.0452 | -0.0178 |
| *EF-4* | 2 | 0.3152 | 0.2949 | -0.0690 |  | 2 | 0.3097 | 0.2886 | -0.0732 |  | 2 | 0.3529 | 0.3652 | 0.0341 |  | 2 | 0.3103 | 0.2794 | -0.1115 |
| *EF-5* | 2 | 0.4982 | 0.4977 | -0.0010 |  | 2 | 0.4516 | 0.4981 | 0.0936 |  | 2 | 0.6176 | 0.5070 | -0.2222 |  | 2 | 0.5349 | 0.4961 | -0.0786 |
| *EF-6* | 3 | 0.6594 | 0.6310 | -0.0452 |  | 3 | 0.6774 | 0.6191 | -0.0946 |  | 3 | 0.6471 | 0.6023 | -0.0756 |  | 3 | 0.6322 | 0.6591 | 0.0411 |
| *EF-7* | 2 | 0.5254 | 0.4946 | -0.0623 |  | 2 | 0.5548 | 0.4924 | -0.1272 |  | 2 | 0.5294 | 0.5057 | -0.0476 |  | 2 | 0.4713 | 0.4933 | 0.0450 |
| *EF-9* | 3 | 0.5181 | 0.4940 | -0.049 |  | 3 | 0.5548 | 0.4961 | -0.1189 |  | 2 | 0.6176 | 0.4965 | -0.2486 |  | 2 | 0.4138 | 0.4949 | 0.1646 |
| *EF-10* | 4 | 0.7862 | 0.7184 | -0.0946 |  | 4 | 0.7871 | 0.7266 | -0.0836 |  | 4 | 0.7647 | 0.7349 | -0.0413 |  | 4 | 0.7931 | 0.7002 | -0.1335 |
| *EF-19* | 3 | 0.4819 | 0.4745 | -0.0156 |  | 2 | 0.4129 | 0.4699 | 0.1216 |  | 2 | 0.3824 | 0.4333 | 0.1191 |  | 3 | 0.6437 | 0.4965 | -0.2986 |
| *EF-20* | 2 | 0.1630 | 0.1739 | 0.0626 |  | 2 | 0.1742 | 0.1595 | -0.0922 |  | 2 | 0.0882 | 0.0856 | -0.0313 |  | 2 | 0.1724 | 0.2307 | 0.2539 |
| *EF-26* | 3 | 0.3732 | 0.3756 | 0.0065 |  | 3 | 0.3742 | 0.3810 | 0.0179 |  | 2 | 0.4118 | 0.3652 | -0.1296 |  | 2 | 0.3563 | 0.3743 | 0.0482 |
| *EF-28* | 6 | 0.6268 | 0.7063 | 0.1127 |  | 6 | 0.6065 | 0.6977 | 0.1311 |  | 5 | 0.6471 | 0.6760 | 0.0435 |  | 6 | 0.6552 | 0.7348 | 0.1089 |
| *EF-29* | 6 | 0.7645 | 0.7743 | 0.0126 |  | 6 | 0.7613 | 0.7667 | 0.0070 |  | 5 | 0.5882 | 0.7441 | 0.2119 |  | 6 | 0.8391 | 0.7934 | -0.0580 |
| *EF-30* | 5 | 0.6486 | 0.6642 | 0.0235 |  | 5 | 0.6452 | 0.6682 | 0.0346 |  | 5 | 0.6471 | 0.6387 | -0.0133 |  | 5 | 0.6552 | 0.6716 | 0.0246 |
| *EF-33* | 10 | 0.8043 | 0.8080 | 0.0045 |  | 10 | 0.8129 | 0.8073 | -0.0069 |  | 6 | 0.7059 | 0.8099 | 0.1301 |  | 7 | 0.8276 | 0.8065 | -0.0263 |
| *EF-35* | 5 | 0.5725 | 0.5186 | -0.1041 |  | 5 | 0.5419 | 0.5151 | -0.0523 |  | 3 | 0.5588 | 0.5009 | -0.1176 |  | 5 | 0.6322 | 0.5370 | -0.1785 |
| *Gm1048* | 6 | 0.7609 | 0.7103 | -0.0713 |  | 6 | 0.7548 | 0.7211 | -0.0469 |  | 6 | 0.7941 | 0.7204 | -0.1041 |  | 5 | 0.7586 | 0.6829 | -0.1115 |
| *Gm2062* | 2 | 0.4457 | 0.4488 | 0.0071 |  | 2 | 0.5032 | 0.4665 | -0.0790 |  | 2 | 0.2353 | 0.4214 | 0.4454 |  | 2 | 0.4253 | 0.4261 | 0.0019 |
| *Gm2024* | 6 | 0.6594 | 0.6231 | -0.0585 |  | 6 | 0.6194 | 0.6262 | 0.0110 |  | 4 | 0.6765 | 0.5948 | -0.1396 |  | 5 | 0.7241 | 0.6270 | -0.1560 |
| *Gm2065* | 7 | 0.7428 | 0.7388 | -0.0054 |  | 7 | 0.7613 | 0.7502 | -0.0149 |  | 6 | 0.7353 | 0.7283 | -0.0098 |  | 7 | 0.7126 | 0.7188 | 0.0086 |
| *Gm4058* | 4 | 0.6884 | 0.6793 | -0.0134 |  | 4 | 0.6710 | 0.6770 | 0.0089 |  | 4 | 0.6765 | 0.6866 | 0.0149 |  | 4 | 0.7241 | 0.6867 | -0.0549 |
|  |  |  |  |  |  |  |  |  |  |  |  |  |  |  |  |  |  |  |  |
|  |  |  |  |  |  |  |  |  |  |  |  |  |  |  |  |  |  |  |  |
|  | **Dinghushan** | | | | | | | | | | | | | | | | | | |
| Locus | All individuals  (33 individuals) | | | |  |  | | | |  |  | | | |  |  | | | |
|  | *A* | *H_O_* | *H_E_* | *f* |  |  |  |  |  |  |  |  |  |  |  |  |  |  |  |
| *EF-1* | 2 | 0.0303 | 0.0303 | − |  |  |  |  |  |  |  |  |  |  |  |  |  |  |  |
| *EF-4* | 2 | 0.4242 | 0.3394 | -0.2549 |  |  |  |  |  |  |  |  |  |  |  |  |  |  |  |
| *EF-5* | 3 | 0.6774 | 0.5394 | -0.2613 |  |  |  |  |  |  |  |  |  |  |  |  |  |  |  |
| *EF-6* | 2 | 0.5152 | 0.4909 | -0.0502 |  |  |  |  |  |  |  |  |  |  |  |  |  |  |  |
| *EF-7* | 2 | 0.5455 | 0.4960 | -0.1013 |  |  |  |  |  |  |  |  |  |  |  |  |  |  |  |
| *EF-9* | 2 | 0.4545 | 0.4909 | 0.0751 |  |  |  |  |  |  |  |  |  |  |  |  |  |  |  |
| *EF-10* | 5 | 0.3636 | 0.4145 | 0.1243 |  |  |  |  |  |  |  |  |  |  |  |  |  |  |  |
| *EF-19* | 3 | 0.3333 | 0.3268 | -0.0203 |  |  |  |  |  |  |  |  |  |  |  |  |  |  |  |
| *EF-20* | 2 | 0.1818 | 0.1678 | -0.0847 |  |  |  |  |  |  |  |  |  |  |  |  |  |  |  |
| *EF-26* | 2 | 0.0303 | 0.0303 | − |  |  |  |  |  |  |  |  |  |  |  |  |  |  |  |
| *EF-28* | 5 | 0.3939 | 0.6746 | 0.4198 |  |  |  |  |  |  |  |  |  |  |  |  |  |  |  |
| *EF-29* | 5 | 0.6970 | 0.7720 | 0.0986 |  |  |  |  |  |  |  |  |  |  |  |  |  |  |  |
| *EF-30* | 4 | 0.3636 | 0.3865 | 0.0600 |  |  |  |  |  |  |  |  |  |  |  |  |  |  |  |
| *EF-33* | 8 | 0.8485 | 0.7837 | -0.0841 |  |  |  |  |  |  |  |  |  |  |  |  |  |  |  |
| *EF-35* | 4 | 0.2727 | 0.3156 | 0.1377 |  |  |  |  |  |  |  |  |  |  |  |  |  |  |  |
| *Gm1048* | 6 | 0.5758 | 0.6284 | 0.0850 |  |  |  |  |  |  |  |  |  |  |  |  |  |  |  |
| *Gm2062* | 2 | 0.3333 | 0.4406 | 0.2463 |  |  |  |  |  |  |  |  |  |  |  |  |  |  |  |
| *Gm2024* | 6 | 0.6667 | 0.6005 | -0.1122 |  |  |  |  |  |  |  |  |  |  |  |  |  |  |  |
| *Gm2065* | 5 | 0.7576 | 0.7212 | -0.0512 |  |  |  |  |  |  |  |  |  |  |  |  |  |  |  |
| *Gm4058* | 4 | 0.7273 | 0.6098 | -0.1963 |  |  |  |  |  |  |  |  |  |  |  |  |  |  |  |

*A*: number of alleles; *H_O_*: observed heterozygosity; *H_E_*: unbiased expected heterozygosity; *f*: fixation index

**P*<0.05 after the Bonferroni correction
