# Supplementary material for: Endangered but genetically stable—Erythrophleum fordii within Feng Shui woodlands in suburbanized villages
Source: Ecol Evol. 2019 Sep 10;9(19):10950–63. doi: 10.1002/ece3.5513 (PMC7277784; doi:10.1002/ece3.5513)
Supplement: Supplementary file 9 [file ECE3-9-10950-s009.docx]

**Table S4** Null allele frequencies with 95% highest posterior density intervals in 20 microsatellite loci for different *Erythrophleum fordii* populations. N: number of individuals

| Locus | TB village  (*N*=346) | WYG village  (*N*=60) | LT village  (*N*=130) | ZPT village  (*N*=164) | ZL village  (*N*=82) | SKY village  (*N*=276) | DH Mountain  (*N*=33) |
| --- | --- | --- | --- | --- | --- | --- | --- |
| *EF-1* | 0.0011  (0-0.0075) | 0.0024  (0-0.0156) | 0.0009  (0-0.0043) | 0.0018  (0-0.0106) | 0.0032  (0-0.0177) | 0.0015  (0-0.01) | 0.0067  (0-0.0433) |
| *EF-4* | 0.0007  (0-0.0039) | 0.0029  (0-0.0163) | 0.0030  (0-0.0216) | 0.0008  (0-0.0041) | 0.0020  (0-0.0121) | 0.0007  (0-0.0031) | 0.0026  (0-0.0146) |
| *EF-5* | 0.0005  (0-0.0023) | 0.0042  (0-0.0299) | 0.0010  (0-0.0064) | 0.0008  (0-0.0059) | 0.0031  (0-0.0164) | 0.0121  (0-0.0481) | 0.0043  (0-0.0251) |
| *EF-6* | 0.0005  (0-0.0024) | 0.0022  (0-0.0147) | 0.0043  (0-0.0294) | 0.0008  (0-0.0038) | 0.0011  (0-0.0069) | 0.0005  (0-0.0024) | 0.0031  (0-0.0183) |
| *EF-7* | 0.0004  (0-0.0022) | 0.0017  (0-0.0089) | 0.0031  (0-0.0203) | 0.0008  (0-0.0041) | 0.0042  (0-0.0299) | 0.0006  (0-0.0037) | 0.0035  (0-0.0228) |
| *EF-9* | 0.0012  (0-0.0070) | 0.0026  (0-0.0151) | 0.0016  (0-0.0097) | 0.0018  (0-0.0116) | 0.0019  (0-0.0114) | 0.0008  (0-0.0051) | 0.0050  (0-0.0273) |
| *EF-10* | 0.0002  (0-0.0017) | 0.0011  (0-0.0067) | 0.0008  (0-0.0047) | 0.0005  (0-0.0023) | 0.0007  (0-0.0027) | 0.0002  (0-0.001) | 0.0047  (0-0.0293) |
| *EF-19* | 0.0004  (0-0.0025) | 0.0013  (0-0.0074) | 0.0009  (0-0.0056) | 0.0004  (0-0.0016) | 0.0010  (0-0.0056) | 0.0009  (0-0.0065) | 0.0057  (0-0.0368) |
| *EF-20* | 0.0017  (0-0.0129) | 0.0042  (0-0.0308) | 0.0026  (0-0.0173) | 0.001  (0-0.0056) | 0.0032  (0-0.0189) | 0.0026  (0-0.0188) | 0.0047  (0-0.0313) |
| *EF-26* | 0.0055  (0-0.0362) | 0.0027  (0-0.0156) | 0.0023  (0-0.0139) | 0.0015  (0-0.0091) | 0.0015  (0-0.0088) | 0.0015  (0-0.0087) | 0.0053  (0-0.0341) |
| *EF-28* | 0.0375  (0-0.0677) | **0.3240**  (0.2267-0.4103) | **0.2149**  (0.1565-0.2695) | **0.1001**  (0.0413-0.1695) | **0.1706**  (0.09-0.2498) | 0.027  (0-0.0656) | **0.0507**  (0-0.1972) |
| *EF-29* | 0.0140  (0-0.0495) | 0.0012  (0-0.0059) | 0.0012  (0-0.007) | 0.0005  (0-0.0029) | 0.0006  (0-0.0027) | 0.0007  (0-0.0039) | 0.0050  (0-0.0344) |
| *EF-30* | 0.0003  (0-0.0012) | 0.0019  (0-0.0111) | 0.0013  (0-0.0069) | 0.0012  (0-0.0061) | 0.0005  (0-0.0028) | 0.0011  (0-0.0066) | 0.0056  (0-0.0375) |
| *EF-33* | 0.0006  (0-0.0031) | 0.0007  (0-0.004) | 0.0005  (0-0.0034) | 0.0004  (0-0.0019) | 0.0003  (0-0.002) | 0.0006  (0-0.0034) | 0.0013  (0-0.0084) |
| *EF-35* | 0.0005  (0-0.0021) | 0.0015  (0-0.0082) | 0.0007  (0-0.0033) | 0.0004  (0-0.0026) | 0.0015  (0-0.0103) | 0.0005  (0-0.0031) | 0.0057  (0-0.0399) |
| *Gm1048* | 0.0004  (0-0.0021) | 0.0012  (0-0.0062) | 0.0006  (0-0.0027) | 0.0005  (0-0.0022) | 0.0004  (0-0.0021) | 0.0003  (0-0.0016) | 0.0054  (0-0.0368) |
| *Gm2062* | 0.0007  (0-0.0043) | 0.0015  (0-0.0087) | 0.0018  (0-0.011) | 0.0033  (0-0.025) | 0.0012  (0-0.0047) | 0.0012  (0-0.0065) | 0.0117  (0-0.0851) |
| *Gm2024* | 0.0004  (0-0.0017) | 0.0128  (0-0.1021) | 0.0079  (0-0.0494) | 0.0035  (0-0.0248) | 0.0014  (0-0.008) | 0.0004  (0-0.0019) | 0.0022  (0-0.0116) |
| *Gm2065* | 0.0003  (0-0.0014) | 0.0007  (0-0.0044) | 0.0004  (0-0.0023) | 0.0003  (0-0.0019) | 0.0007  (0-0.0026) | 0.0004  (0-0.0021) | 0.0024  (0-0.0144) |
| *Gm4058* | 0.0008  (0-0.0047) | 0.0053  (0-0.0336) | 0.0008  (0-0.005) | 0.0013  (0-0.0091) | 0.0007  (0-0.0032) | 0.0005  (0-0.0023) | 0.0019  (0-0.0108) |
