# Supplementary material for: Endangered but genetically stable—Erythrophleum fordii within Feng Shui woodlands in suburbanized villages
Source: Ecol Evol. 2019 Sep 10;9(19):10950–63. doi: 10.1002/ece3.5513 (PMC7277784; doi:10.1002/ece3.5513)
Supplement: Supplementary file 10 [file ECE3-9-10950-s010.docx]

**Table S5** One-sided (the left population value less or greater than the right population value) P-values for the non-parametric Wilconxon tests of differences in observed heterozygosity (*H*_O_), expected heterozygosity (*H*_E_), allelic richness (*A*_R_) and private allelic richness (*A*_P_)

| Populations | *H_O_* | |  | *H*_E_ | |  | *A_R_* | |  | *A_P_* | |
| --- | --- | --- | --- | --- | --- | --- | --- | --- | --- | --- | --- |
|  | Less | Greater |  | Less | Greater |  | Less | Greater |  | Less | Greater |
| **TB-WYG** | 0.6036 | 0.4077 |  | 0.8993 | 0.1060 |  | 0.8704 | 0.1347 |  | 0.9105 | 0.0934 |
| **TB-LT** | 0.5349 | 0.4767 |  | 0.4200 | 0.5913 |  | 0.5607 | 0.4486 |  | 0.8333 | 0.1727 |
| **TB-ZPT** | 0.4767 | 0.5349 |  | 0.5573 | 0.4541 |  | 0.5374 | 0.4719 |  | 0.8273 | 0.1787 |
| **TB-ZL** | 0.2796 | 0.7301 |  | 0.3976 | 0.6135 |  | 0.6808 | 0.3276 |  | 0.8661 | 0.1391 |
| **TB-SKY** | 0.1905 | 0.8173 |  | 0.2261 | 0.7825 |  | 0.4254 | 0.5838 |  | 0.6664 | 0.3421 |
| **TB-DH** | 0.6902 | 0.3202 |  | 0.7105 | 0.2996 |  | 0.6467 | 0.3621 |  | 0.3086 | 0.6996 |
| **WYG-LT** | 0.4076 | 0.6037 |  | 0.1354 | 0.8709 |  | 0.1767 | 0.8293 |  | 0.2766 | 0.7312 |
| **WYG-ZPT** | 0.3521 | 0.6587 |  | 0.1485 | 0.8582 |  | 0.1451 | 0.8602 |  | 0.2389 | 0.7683 |
| **WYG-ZL** | 0.1905 | 0.8173 |  | 0.0578 | 0.9455 |  | 0.2054 | 0.8012 |  | 0.4308 | 0.5783 |
| **WYG-SKY** | 0.1905 | 0.8173 |  | **0.0186** | 0.9828 |  | 0.0981 | 0.9059 |  | 0.1057 | 0.8985 |
| **WYG-DH** | 0.6037 | 0.4076 |  | 0.2796 | 0.7301 |  | 0.2019 | 0.8047 |  | **0.0423** | 0.9599 |
| **LT-ZPT** | 0.5465 | 0.4651 |  | 0.6024 | 0.4087 |  | 0.4486 | 0.5607 |  | 0.4400 | 0.5692 |
| **LT-ZL** | 0.2327 | 0.7762 |  | 0.5000 | 0.5115 |  | 0.5974 | 0.4117 |  | 0.6914 | 0.3168 |
| **LT-SKY** | 0.2327 | 0.7761 |  | 0.2532 | 0.7560 |  | 0.4026 | 0.6065 |  | 0.3086 | 0.6996 |
| **LT-DH** | 0.7491 | 0.2603 |  | 0.7583 | 0.2509 |  | 0.5700 | 0.4393 |  | 0.1339 | 0.8711 |
| **ZPT-ZL** | 0.2747 | 0.7350 |  | 0.4313 | 0.5800 |  | 0.6808 | 0.3276 |  | 0.6664 | 0.3421 |
| **ZPT-SKY** | 0.2417 | 0.7673 |  | 0.2372 | 0.7717 |  | 0.4254 | 0.5838 |  | 0.3004 | 0.7076 |
| **ZPT-DH** | 0.7301 | 0.2796 |  | 0.6850 | 0.3254 |  | 0.6335 | 0.3754 |  | 0.1727 | 0.8333 |
| **ZL-SKY** | 0.5233 | 0.4884 |  | 0.3325 | 0.6779 |  | 0.2784 | 0.7294 |  | 0.1443 | 0.8609 |
| **ZL-DH** | 0.8954 | 0.1100 |  | 0.7890 | 0.2195 |  | 0.4672 | 0.5421 |  | 0.0934 | 0.9105 |
| **SKY-DH** | 0.8900 | 0.1156 |  | 0.9150 | 0.0896 |  | 0.7098 | 0.2983 |  | 0.2317 | 0.7753 |

The numbers in bold are *P* < 0.05; refer to Table 1 in text for the population names.
