# Supplementary material for: Endangered but genetically stable—Erythrophleum fordii within Feng Shui woodlands in suburbanized villages
Source: Ecol Evol. 2019 Sep 10;9(19):10950–63. doi: 10.1002/ece3.5513 (PMC7277784; doi:10.1002/ece3.5513)
Supplement: Supplementary file 11 [file ECE3-9-10950-s011.docx]

**Table S6** One-sided (the left life stage value less or greater than the right life stage value) P-values for the non-parametric Wilconxon tests of differences in observed heterozygosity (*H*_O_), expected heterozygosity (*H*_E_), allelic richness (*A*_R_) and private allelic richness (*A*_P_)

| Populations | *H_O_* value | |  | *H_E_* value | |  | Allelic richness | |  | Private allelic richness | |
| --- | --- | --- | --- | --- | --- | --- | --- | --- | --- | --- | --- |
|  | Less | Greater |  | Less | Greater |  | Less | Greater |  | Less | Greater |
| **TB village** |  |  |  |  |  |  |  |  |  |  |  |
| Adult - Juvenile | 0.4535 | 0.5581 |  | 0.5407 | 0.4709 |  | 0.5669 | 0.4419 |  | 0.5606 | 0.4486 |
| Adult - Seedling | 0.5924 | 0.4190 |  | 0.6479 | 0.3630 |  | 0.8133 | 0.1928 |  | 0.7630 | 0.2443 |
| Juvenile - Seedling | 0.5810 | 0.4305 |  | 0.5345 | 0.4770 |  | 0.8190 | 0.1868 |  | 0.6551 | 0.3536 |
| **WYG village** |  |  |  |  |  |  |  |  |  |  |  |
| Adult+Juvenile - Seedling | 0.5639 | 0.4477 |  | 0.7204 | 0.2895 |  | 0.3935 | 0.6155 |  | 0.0565 | 0.9461 |
| **LT village** |  |  |  |  |  |  |  |  |  |  |  |
| Adult+Juvenile - Seedling | 0.6149 | 0.3963 |  | 0.5116 | 0.5000 |  | 0.5468 | 0.4625 |  | 0.6579 | 0.3507 |
| **ZPT village** |  |  |  |  |  |  |  |  |  |  |  |
| Adult - Juvenile | 0.5349 | 0.4767 |  | 0.3852 | 0.626 |  | 0.3535 | 0.6552 |  | 0.2389 | 0.7683 |
| Adult - Seedling | 0.3630 | 0.6479 |  | 0.2417 | 0.7673 |  | 0.4026 | 0.6064 |  | 0.3004 | 0.7076 |
| Juvenile - Seedling | 0.2746 | 0.7350 |  | 0.4651 | 0.5465 |  | 0.6244 | 0.3845 |  | 0.5600 | 0.4492 |
| **ZL village** |  |  |  |  |  |  |  |  |  |  |  |
| Adult - Juvenile+Seedling | 0.4767 | 0.5349 |  | 0.3740 | 0.6370 |  | 0.3069 | 0.7013 |  | 0.3406 | 0.6679 |
| **SKY village** |  |  |  |  |  |  |  |  |  |  |  |
| Adult - Juvenile | 0.5695 | 0.4420 |  | 0.5349 | 0.4767 |  | 0.6519 | 0.3569 |  | 0.0918 | 0.0857 |
| Adult - Seedling | 0.4076 | 0.6037 |  | 0.5058 | 0.5058 |  | 0.4953 | 0.5141 |  | 0.4954 | 0.5139 |
| Juvenile - Seedling | 0.2796 | 0.7301 |  | 0.5523 | 0.4593 |  | 0.3837 | 0.6253 |  | 0.1290 | 0.8760 |
